# Interplay between Mutations and Efflux in Drug Resistant Clinical Isolates of *Mycobacterium tuberculosis*

**DOI:** 10.3389/fmicb.2017.00711

**Published:** 2017-04-27

**Authors:** Diana Machado, Tatiane S. Coelho, João Perdigão, Catarina Pereira, Isabel Couto, Isabel Portugal, Raquel De Abreu Maschmann, Daniela F. Ramos, Andrea von Groll, Maria L. R. Rossetti, Pedro A. Silva, Miguel Viveiros

**Affiliations:** ^1^Unidade de Microbiologia Médica, Global Health and Tropical Medicine, Instituto de Higiene e Medicina Tropical, Universidade Nova de LisboaLisboa, Portugal; ^2^Programa de Pós-Graduação em Biologia Celular e Molecular, Centro de Biotecnologia, Fundação Universidade Federal do Rio GrandePorto Alegre, Brazil; ^3^Núcleo de Pesquisa em Microbiologia Médica, Faculdade de Medicina, Fundação Universidade Federal do Rio GrandePorto Alegre, Brazil; ^4^iMed.ULisboa, Instituto de Investigação do Medicamento, Faculdade de Farmácia, Universidade de LisboaLisboa, Portugal; ^5^Fundação Estadual de Produção e Pesquisa em Saúde, Centro de Desenvolvimento Científico e TecnológicoPorto Alegre, Brazil; ^6^Programa de Pós-Graduação em Biologia Celular e Molecular Aplicada à Saúde, Universidade Luterana do BrasilCanoas, Brazil

**Keywords:** tuberculosis, synergism, efflux inhibitors, TB eXIST, time to detection

## Abstract

Numerous studies show efflux as a universal bacterial mechanism contributing to antibiotic resistance and also that the activity of the antibiotics subject to efflux can be enhanced by the combined use of efflux inhibitors. Nevertheless, the contribution of efflux to the overall drug resistance levels of clinical isolates of *Mycobacterium tuberculosis* is poorly understood and still is ignored by many. Here, we evaluated the contribution of drug efflux plus target-gene mutations to the drug resistance levels in clinical isolates of *M. tuberculosis*. A panel of 17 *M. tuberculosis* clinical strains were characterized for drug resistance associated mutations and antibiotic profiles in the presence and absence of efflux inhibitors. The correlation between the effect of the efflux inhibitors and the resistance levels was assessed by quantitative drug susceptibility testing. The bacterial growth/survival vs. growth inhibition was analyzed through the comparison between the time of growth in the presence and absence of an inhibitor. For the same mutation conferring antibiotic resistance, different MICs were observed and the different resistance levels found could be reduced by efflux inhibitors. Although susceptibility was not restored, the results demonstrate the existence of a broad-spectrum synergistic interaction between antibiotics and efflux inhibitors. The existence of efflux activity was confirmed by real-time fluorometry. Moreover, the efflux pump genes *mmr, mmpL7, Rv1258c, p55*, and *efpA* were shown to be overexpressed in the presence of antibiotics, demonstrating the contribution of these efflux pumps to the overall resistance phenotype of the *M. tuberculosis* clinical isolates studied, independently of the genotype of the strains. These results showed that the drug resistance levels of multi- and extensively-drug resistant *M. tuberculosis* clinical strains are a combination between drug efflux and the presence of target-gene mutations, a reality that is often disregarded by the tuberculosis specialists in favor of the almost undisputed importance of antibiotic target-gene mutations for the resistance in *M. tuberculosis*.

## Introduction

The development of mutations in the genes associated with the resistance to the antituberculosis drugs have long been considered the sole cause of resistance in tuberculosis (Zhang and Yew, 2009; da Silva and Palomino, 2011). Nevertheless, *Mycobacterium tuberculosis* also presents intrinsic drug resistance, mainly attributed to the unusual structure of its mycolic acid-containing cell wall combined with effective efflux mechanisms (Jarlier and Nikaido, 1994; da Silva et al., 2011). The balance between the reduced permeability of the cell wall that acts synergistically with the activity of efflux pumps and the increased expression of genes that code for those efflux pumps is believed to constitute the first step for the development and stabilization of drug resistant phenotypes (Machado et al., 2012; Schmalstieg et al., 2012; Viveiros et al., 2012; da Silva et al., 2016).

Previous studies have demonstrated the contribution of efflux mechanisms to antibiotic resistance in *M. tuberculosis* revealing the presence of several putative efflux pumps of different classes involved in the transport of different compounds (Viveiros et al., 2012; Black et al., 2014; da Silva et al., 2016; Supplementary Table 1). The best-represented families of efflux transporters in *M. tuberculosis* are the ATP-binding cassette (ABC) superfamily and the major facilitator superfamily (MFS) followed by the resistance nodulation cell division (RND) superfamily of transporters. The most well-characterized ABC transporters showed to be involved in the transport of multiple drugs are the efflux pumps DrrAB, Rv2686c-2687c-2688c, Rv1456c-Rv1457c-Rv1458c, and the Rv1217c-1218c (Choudhuri et al., 2002; Pasca et al., 2004; Balganesh et al., 2010; Hao et al., 2011; Wang et al., 2013). Among the MFS, the most studied efflux pumps are the Rv1258c (Tap-like), associated with resistance to tetracycline, rifampicin and clofazimine (Siddiqi et al., 2004; Ramón-García et al., 2012); the P55, that confer resistance to aminoglycosides, tetracycline, rifampicin and clofazimine (da Silva et al., 2001; Ramón-García et al., 2009; Bianco et al., 2011); and the EfpA efflux transporter that is associated with resistance to isoniazid, fluoroquinolones and dyes (Doran et al., 1997). The RND efflux pumps are associated with the transport of a wide variety of substrates in *M. tuberculosis* (Bailo et al., 2015). Among these, it was shown that the MmpL7 protein confer low-level isoniazid resistance when overexpressed (Pasca et al., 2005; Machado et al., 2012). Likewise, the overexpression of the MmpS5-MmpL5 efflux transporter was associated with the resistance of *M. tuberculosis* to azoles (Milano et al., 2009) and with the acquired resistance to bedaquiline, the diarylquinoline recently approved for the treatment of multidrug resistant tuberculosis (Andries et al., 2014). The Mmr efflux transporter is the only efflux pump from the small multidrug resistance (SMR) family present in the *M. tuberculosis* genome and is associated with the reduced susceptibility of *M. tuberculosis* to dyes and antibiotics such as isoniazid, erythromycin, and fluoroquinolones (De Rossi et al., 1998; Rodrigues et al., 2013).

The overexpression of these and other efflux pumps is believed to decrease the intracellular levels of the antibiotics and prevent the drug to reach its cellular target, allowing the emergence of a subpopulation presenting high-level resistance (Machado et al., 2012; Schmalstieg et al., 2012). Noteworthy, efflux-based mechanisms were recently implicated in the intrinsic resistance to bedaquiline, and the efflux system MmpS5-MmpL5 was identified as being responsible for the non-target based resistance (Andries et al., 2014). In this sense, an approach to overcome the emergence of this efflux-mediated drug resistance can be the combined use of efflux inhibitors and antibiotics.

Up to now, a number of compounds have been shown as potential inhibitors of the *M. tuberculosis* efflux systems (Viveiros et al., 2012; da Silva et al., 2016). Among these, the most studied are the ion channel blockers like the phenothiazines thioridazine and chlorpromazine and the phenylalkylamine verapamil (Viveiros et al., 2012; da Silva et al., 2016; Machado et al., 2016). The mechanism by which these compounds are active against *M. tuberculosis* is not fully understood, however, some hypothesis for their mode action have been raised and discussed (Viveiros et al., 2012; Adams et al., 2014; Rayasam and Balganesh, 2015). Verapamil, a calcium channel blocker in eukaryotic cells, is able to decrease the minimum inhibitory concentrations (MICs) of antituberculosis drugs in *M. tuberculosis* clinical strains (Louw et al., 2011; Machado et al., 2012; Coelho et al., 2015), accelerates the bactericidal and sterilizing activities of antituberculosis drugs in a mouse model (Gupta et al., 2013), potentiates the activity of bedaquiline (Gupta et al., 2014), and inhibits macrophage-induced antibiotic tolerance of *M. tuberculosis* (Adams et al., 2011, 2014). Likewise, the phenothiazines have shown similar activities in spite of their toxicity compared to verapamil and other efflux inhibitors (Ordway et al., 2003; Viveiros et al., 2012). The phenothiazines disturb the calcium-calmodulin transport and signaling pathways (Salih et al., 1991; Pluta et al., 2011), cause modifications at the level of the bacterial membrane and on nucleic acid stability (Pluta et al., 2011; Thorsing et al., 2013), inhibit the type II NADH-ubiquinone dehydrogenase and other electron-transport chain enzymes (Weinstein et al., 2005), and have significant activity against *M. tuberculosis in vitro, ex vivo*, and in murine models (Ordway et al., 2003; Machado et al., 2012; Dutta et al., 2014; Singh and Sharma, 2014; Coelho et al., 2015). Recently, we observed that these ion channel blockers lead to a significant decrease in the intracellular mycobacterial load as result of the inducement of phagosome acidification and activation of lysosomal hydrolases (Machado et al., 2016). The potential use of efflux inhibitors in combination with antibiotics may constitute an important alternative as adjuvants of the antituberculosis conventional therapeutic regimen. Previously, we have demonstrated that multidrug resistance and in particular the development of resistance to isoniazid develops in *M. tuberculosis* laboratory strains associated with the genetic and physiological activity of efflux pumps (Machado et al., 2012). Nonetheless, two questions remain answered—the role of the efflux systems in *M. tuberculosis* resistant strains that already carry mutations in the drug target genes and how do they respond to the drug pressure despite the presence of these mutations—a contribution to the overall resistance level poorly understood and still disregarded by many.

The main purpose of this study is to assess the role played by the efflux systems in *M. tuberculosis* strains that carry mutations in the drug target genes. In order to do this, we have studied the contribution of efflux to the overall resistance levels toward antituberculosis drugs in a panel of mono-, multi- and extensively drug resistant (MDR/XDR) *M. tuberculosis* clinical isolates from two geographical distinct areas baring well-known and characterized drug-resistance associated mutations. To quantify the role of efflux in these strains and establish clinical correlation, we performed quantitative drug susceptibility testing (qDST) and investigated the time to detection (TTD) of growth of each strain for first- and second-line antituberculosis drugs in the presence and absence of the efflux inhibitors verapamil, thioridazine, and chlorpromazine. The results obtained revealed that the addition of efflux inhibitors enhanced the effect of the antituberculosis drugs independently of the genotype of the *M. tuberculosis* strains. Here we have demonstrated the effectiveness of a synergistic combination of drugs with the conventional therapy despite the presence of a mutation conferring resistance that showed to be beneficial since it reduced the resistance level of the strain. Moreover, these results strongly support the relevance of these inhibitors as adjuvants in tuberculosis chemotherapy.

## Materials and methods

### Strains

The strains selected for this study are described in Table 1 and were isolated from Portuguese and Brazilian tuberculosis patients as part of the routine mycobacteriology laboratory services provided by Universidade NOVA de Lisboa (Lisboa, Portugal) and Universidade Federal do Rio Grande (Porto Alegre, Brazil) to the local hospitals. Since the main objective of this work was to analyze the interplay between the mutational resistance and the efflux activity, the strains were selected based on their drug resistance pattern in order to include pan-susceptible, isoniazid monoresistant, rifampicin monoresistant, ofloxacin monoresistant, poly-drug resistant (resistant to more than one first-line antituberculosis drugs other than both isoniazid and rifampicin), MDR (resistant at least to isoniazid and rifampicin), and XDR (MDR plus resistance to any fluoroquinolone and amikacin, kanamycin, or capreomycin) strains. The H37Rv ATCC27294^T^ reference strain was obtained from the American Type Culture Collection (Virginia, USA), and used as control. Given the retrospective nature of the work involving only anonymized bacterial isolates, informed consent was not required for this study.

**Table 1:** **Categorization of the *M. tuberculosis* strains studied according to their lineage, resistance pattern, and phenotype**.

**Strain**	**Isolation date**	Lineage^*^	**Resistance pattern**	**Phenotype**
H37Rv	–	H37Rv	None	Susceptible
MtbPT1	2006	LAM9—Orphan	None	Susceptible
MtbPT2	2008	Unknown—SIT150	None	Susceptible
MtbPT3	2003	T1	INH	Monoresistant to INH
MtbPT4	2009	LAM1	INH	Monoresistant to INH
MtbPT5	2003	LAM1	RIF	Monoresistant to RIF
MtbPT6	2005	LAM1	RIF	Monoresistant to RIF
MtbBR1	2009	EAI1-SOM	OFX	Monoresistant to OFX
MtbBR2	2007	Beijing	INH, OFX	Poly-drug resistant
MtbBR3	2010	H3	INH, RIF	MDR
MtbBR4	2011	T1	INH, RIF	MDR
MtbBR5	2011	T2	INH, RIF	MDR
MtbPT7	2009	LAM1—Lisboa3^&^	INH; RIF	MDR
MtbBR6	2008	Beijing	INH, RIF, AMK, CAP	MDR
MtbPT8	2011	Beijing	INH; RIF; AMK; CAP	MDR
MtbPT9	2009	LAM1—Lisboa3^&^	INH; RIF; AMK; CAP; OFX	XDR
MtbPT10	2009	LAM4—Q1^&^	INH; RIF; AMK; CAP; OFX	XDR
MtbPT11	2012	LAM4—Q1^&^	INH; RIF; AMK; CAP; OFX	XDR

* Spoligotype lineages/sublineages according to the international spoligotype database SITVITWEB rules. “Unknown” designates patterns with signatures that do not belong to any of the major lineages defined in the SITVITWEB database.

& *Lisboa3 and Q1 M. tuberculosis strains were clustered by 24-loci MIRU-VNTR analysis. AMK, amikacin; CAP, capreomycin; EAI, East-African-Indian; H, Haarlem. INH, isoniazid; LAM, Latin American-Mediterranean; MDR, multidrug resistant; OFX, ofloxacin; RIF, rifampicin; SIT, spoligotype international type; XDR, extensively drug resistant*.

### Drug susceptibility testing and MIC determination

First- and second-line drug susceptibility testing (DST) and MIC determination were done using the MGIT 960 system using the Epicenter V5.80A software and the TB eXIST module (Becton Dickinson, Diagnostic Systems, Sparks, MD, USA). The critical concentrations used for DST were as follows: isoniazid, 0.1 μg/ml; rifampicin, 1 μg/ml; streptomycin, 1 μg/ml; ethambutol, 5 μg/ml; amikacin, 1 μg/ml; capreomycin, 2.5 μg/ml; and ofloxacin, 1 μg/ml. The results were interpreted as follows: at the time of positivity of the proportional control [Growth units (GU) = 400] if the tubes containing the drugs were GU > 100, they were considered resistant to that concentration. If the GU of the tube containing the drugs were <100 they were considered susceptible (Springer et al., 2009; Cambau et al., 2015). In these assays, the critical concentration of the antituberculosis drugs are defined as the concentration that is required to eliminate more than 99% of the population of a control strain that never has been in contact with the drug. A strain is considered susceptible to a given drug tested at their critical concentration if, among the population, the proportion of resistant cells is <1% (critical proportion). The strain is considered resistant when the number of drug-resistant bacteria present in the drug-containing tube is >1%, when compared with the drug-free proportional growth control.

MIC determination of the antibiotics and efflux inhibitors was done within the MGIT 960 system and the growth monitored with the Epicenter V5.80A software as previously described (Machado et al., 2016). The MIC was considered as the lowest concentration with GU < 100 when the drug-free proportional control tube reached a GU = 400. The lowest concentration tested corresponded to the critical concentration of each antibiotic (susceptibility breakpoint).

An absolute growth control (undiluted) was included in every assay to monitor the normal growth of each strain. In this study, the TTD for positivity of the drug-free proportional control varied between 8 and 16 days depending on the strains. DST and MIC determination were performed at least in duplicate and the final value was given as the result of two concordant values.

### Quantitative drug susceptibility testing

qDST of the antibiotics was conducted using the MGIT 960 system and the growth monitored with the Epicenter V5.80A software and the TB eXIST module (Becton Dickinson). The test was performed and interpreted according to the protocol previously described (Cambau et al., 2015). Isoniazid was tested at 0.1, 1, 3, and 10 μg/ml, rifampicin and amikacin at 1, 4, and 20 μg/ml, capreomycin at 2.5, 5, and 25 μg/ml, and ofloxacin at 1, 2, and 10 μg/ml. Antibiotics for which the strains were resistant at the highest concentration tested by qDST, were further tested at higher concentrations by MIC determination. The interpretation of the results was done as described above. The antibiotic resistance levels were quantified as follows: isoniazid low-level resistance when resistant (R) at 0.1 and susceptible (S) at 1 μg/ml; isoniazid high-level resistance when R ≥ 1 μg/ml; rifampicin and amikacin low-level resistance when R at 4 and S at 20 μg/ml; rifampicin and amikacin high-level resistance when R ≥ 20 μg/ml; capreomycin low-level resistance when R at 5 and S at 25 μg/ml; capreomycin high-level resistance when R ≥ 25 μg/ml; ofloxacin low-level resistance when R at 1 and S at 2 μg/ml; ofloxacin high-level resistance when R ≥ 2 μg/ml (Cambau et al., 2015). The evaluation of bacterial growth/survival vs. growth inhibition was done by the comparison between the TTD, defined as the time from the start of incubation to the positivity signal of growth (Diacon et al., 2014; Bowness et al., 2015), of a given strain in the presence and absence of an inhibitor (ΔTTD). To obtain the degree of potentiation of each compound on the antibiotic activity, the ΔTTD was normalized against the control tube with no inhibitor. qDST was performed at least in duplicate and the final value was given as the result of two concordant values.

### Detection of mutations associated with resistance

Genomic DNA was isolated using the QIAamp DNA mini kit (QIAGEN, GmbH, Hilden, Germany) according to the manufacturer's instructions. The most common mutations in *inhA* regulatory region, and *katG, gyrA*, and *rrs* genes were studied using the Genotype MTBDR*plus* and MTBDR*sl* (Hain Lifescience GmbH, Nehren, Germany) according to the manufacturer's instructions. Genomic analysis of the complete *inhA, katG*, and *tlyA* genes and *eis* promoter region was performed by PCR amplification and DNA sequencing using the primers described elsewhere (Feuerriegel et al., 2009; Machado et al., 2013; Perdigão et al., 2013). The annealing temperatures were 60°C for *inhA*, 62°C for *katG*, 56°C for *eis*, and 55°C for *tlyA*.

### Evaluation of ethidium bromide accumulation and efflux

Real-time detection of efflux activity was performed with a semi-automated fluorometric method using a Rotor-Gene 3000 thermocycler (Corbett Research, Sydney, Australia) (Paixão et al., 2009; Viveiros et al., 2010; Rodrigues et al., 2015). The assays were performed as previously described (Machado et al., 2012). *M. tuberculosis* strains were grown in 10 ml of Middlebrook 7H9 (Difco, Madrid, Spain) supplemented with 10% OADC (oleic acid, albumin, dextrose, catalase; Becton and Dickinson) with 0.05% Tween 80 at 37°C until an OD_600 *nm*_(OD_600_) of 0.8. The cells were collected by centrifugation at 2940 × g for 3 min, the supernatant discarded, the pellet washed in PBS and centrifuged again in the same conditions.

#### Ethidium bromide accumulation assays

For the ethidium bromide accumulation assays, the pellet was resuspended in PBS and the OD_600_ adjusted to 0.8. To determine the concentration of ethidium bromide that establishes the equilibrium between efflux and influx, the cells were incubated with different concentrations of ethidium bromide (0.625–5 μg/ml). The assays were conducted at 37°C in a Rotor-Gene 3000 (530; 585 nm) and the equilibrium concentration determined for each strain as the concentration that promoted a plateau of no more than 10% of relative fluorescence units during the 60 min of the assay. To evaluate the effect of the efflux inhibitors on the accumulation of ethidium bromide, the assays were performed as described above with the exception that each inhibitor was added to the buffer solution at half MIC and ethidium bromide was used at the equilibrium concentration (determined for each strain). The inhibitory activity of the compounds was determined by the calculation of the relative final fluorescence (RFF; Machado et al., 2011) which is a measure of how effective the compound is on the inhibition of ethidium bromide efflux (at a given concentration) by comparison of the final fluorescence at the last time point (60 min) of the treated cells with the cells in presence of only ethidium bromide. An index of activity above zero indicated that the cells accumulate more ethidium bromide under the condition used than those of the control (non-treated cells). Negative RFF values indicated that the treated cells accumulated less ethidium bromide than those of the control condition. Values above 1 in the presence of the efflux inhibitors indicated enhanced accumulation of ethidium bromide inside the cells. The experiments were done in triplicate and the RFF values are presented as the average of three independent assays plus standard deviation (±*SD*).

#### Ethidium bromide efflux assays

For the ethidium bromide efflux assays, the pellet was resuspended in PBS and the OD_600_ adjusted to 0.4. The cell suspensions were exposed to ethidium bromide at the equilibrium concentration determined above, in the presence of verapamil at half MIC for 1 h at room temperature. The cells were then pelleted by centrifugation and resuspended in new buffer with and without 0.4% glucose in the presence and absence of half MIC of verapamil and the fluorescence measured as described above. Data was normalized by comparing the relative fluorescence units obtained under the conditions that promote efflux (presence of glucose and absence of verapamil) with the relative fluorescence units from the control in which there is no efflux (presence of verapamil and no glucose). The RFF corresponds to the ratio of the fluorescence that remains per unit of time, relatively to the cells in presence of the inhibitor.

### RT–qPCR analysis of putative efflux pumps

Strains were grown in MGIT tubes (mycobacteria growth indicator tubes; Becton Dickinson) for the MGIT 960 system in the presence of half MIC of each antibiotic as follows: MtbPT3 was exposed to isoniazid; MtbPT5 was exposed to rifampicin; MtbBR1 was exposed to ofloxacin; H37Rv, MtbPT7, and MtbPT11 were exposed to isoniazid or rifampicin. Total RNA was extracted using a GTC/Trizol method as previously described (Machado et al., 2016). The relative expression level of the efflux pump genes *mmpL7, p55, efpA, mmr, Rv1258c*, and *Rv2459* was analyzed by RT-qPCR. The primer sets and sequences of oligonucleotides used are described elsewhere (Machado et al., 2012). RT-qPCR assay was performed in a Rotor-Gene 3000 thermocycler and followed the protocol recommended for use with the QuantiTect SYBR Green RT-PCR Kit (QIAGEN) with the following amplification program: reverse transcription for 30 min at 50°C; initial activation step for 15 min at 95°C; 35 cycles of denaturation at 94°C for 30 s, annealing at 52°C for 30 s and extension at 72°C for 30 s; a final extension step at 72°C for 5 min; and an additional step at 50°C for 15 s followed by melt analysis (50–99°C). The determination of the relative mRNA expression level was performed using the comparative quantification cycle (Cq) method (Livak and Schmittgen, 2001). The relative expression of each target gene was determined by comparison of the relative quantity of the respective mRNA in the presence of the antibiotic to the non-exposed condition. Each strain was assayed in triplicate using total RNA obtained from three independent cultures. A relative expression level equal to 1 indicated that the expression level was identical to the unexposed strain. Genes showing expression levels above one were considered to be overexpressed. Genes showing expression levels above two were considered to be significantly overexpressed. Normalization of the mRNA levels was done using the *M. tuberculosis* 16S rDNA as internal control for each experiment and presented as the mean-fold change (±*SD*) compared with the control.

### MIRU-VNTR and spoligotyping analysis

Mycobacterial Interspersed Repetitive Unit—Variable Number of Tandem Repeats (MIRU-VNTR) genotyping was performed by multiplex PCR amplification of 24-*loci* MIRU-VNTR, as described previously (Supply et al., 2006). Spoligotyping was performed as per Kamerbeek et al. (1997). The genotype of these strains was analyzed using the MIRU-VNTR*plus* web application (Allix-Béguec et al., 2008; Weniger et al., 2010) and the SITVITWEB database (Demay et al., 2012).

### Data analysis

Data analysis was carried out using the Student's *t*-test. A ^*^P < 0.05 was considered statistically significant and highly significant when ^**^P < 0.01 and ^***^P < 0.001 (two-tailed tested).

## Results

### Correlation between the phenotype and mutations associated with antibiotic resistance

Firstly, to establish a correlation between the phenotypic drug resistance level and the presence of resistance associated mutations, we searched for mutations in the genes associated with drug resistance to the antibiotics studied (Table 2).

**Table 2:** **Mutations associated with resistance to first- and second-line drugs detected in the *M. tuberculosis* strains in study**.

**Strain ID**	**Mutations**							
	**INH**			**RIF**	**AMK/CAP**	**AMK**	**CAP**	**OFX**
	***inhA* prom**	***inhA* ORF**	***katG***	***rpoB* RRDR**	***rrs* 1400 region**	***eis* prom**	***tlyA***	***gyrA* QRDR**
**SUSCEPTIBLE**								
H37Rv	wt	wt	wt	wt	wt	wt	wt	wt
MtbPT1	wt	wt	wt	wt	wt	wt	wt	wt
MtbPT2	wt	wt	wt	wt	wt	wt	wt	wt
**INH^R^**								
MtbPT3	wt	wt	S315T	wt	wt	nt	nt	wt
MtbPT4	C-15T	wt	wt	wt	wt	nt	nt	wt
**RIF^R^**								
MtbPT5	wt	nt	wt	S531L	wt	wt	wt	wt
MtbPT6	wt	nt	wt	S531L	wt	nt	nt	wt
**OFX^R^**								
MtbBR1	wt	nt	wt	wt	wt	nt	nt	D94N
**Poly-DR**								
MtbBR2	wt	nt	S315T	D516Y	wt	nt	nt	A90V
**MDR**								
MtbBR3	wt	nt	S315T	S531L	wt	wt	nt	wt
MtbBR4	wt	nt	S315T	S531L	wt	wt	nt	wt
MtbBR5	wt	nt	S315T	S531L	wt	wt	nt	wt
MtbPT7	C-15T	S94A	wt	S531L	wt	wt	wt	wt
MtbBR6	wt	nt	D735A	S531L	A1401G	wt	wt	wt
MtbPT8	wt	wt	S315T	S531L	wt/A1401G^*^	wt	wt	wt
**XDR**								
MtbPT9	C-15T	S94A	wt	S531L	wt	G-10A	ins GT at pos 755/756	S91P
MtbPT10	C-15T	I194T	wt	S531L	A1401G	wt	wt	D94A
MtbPT11	C-15T	I194T	wt	S531L	A1401G	nt	nt	D94A

* *Heteroresistance. AMK, amikacin; CAP, capreomycin; ID, identification; INH, isoniazid; MDR, multidrug resistant; nt, not tested; OFX, ofloxacin; ORF, open reading frame; Poly-DR, poly-drug resistant; Prom, promoter; QRDR, quinolone resistance determining region; RIF, rifampicin; RRDR, rifampicin resistance determining region; wt, wild-type sequence; XDR, extensively drug resistant*.

The three pan-susceptible strains showed no mutation in any of the genes evaluated. Resistance to isoniazid was associated with mutations in the *katG* and *inhA* genes which are in accordance with published data (Cambau et al., 2015; Domínguez et al., 2016). MtbPT3 showed the S315T mutation in *katG* and MtbPT4 presented the C-15T substitution in the *inhA* promoter region. Among the nine MDR strains, five harbored mutations in *katG*, of which four had the S315T mutation and one has a rare mutation at codon 735. The remaining four strains harbored double mutations in *inhA*. The double mutations C-15T/S94A were detected in strains MtbPT7 and MtbPT9 and the pair C-15T/I194T was detected in MtbPT10 and MtbPT11.

In respect to rifampicin resistance, all MDR strains and the two rifampicin monoresistant strains showed the mutation S531L in *rpoB*. This is the most common mutation found in clinical isolates resistant to rifampicin (Cambau et al., 2015; Domínguez et al., 2016). Strain MtbBR2 carries the mutation D516Y in *rpoB* but it was found susceptible to rifampicin. Although was not our aim to evaluate susceptible strains, MtbBR2 was initially found to be resistant to rifampicin at 1 μg/ml (critical concentration). After re-testing MtbBR2 for rifampicin resistance, it was found susceptible, although with a MIC close to the breakpoint, and was reclassified as poly-drug resistant. The role of the mutation D516Y in rifampicin resistance is not clear. It has been reported by others to be associated with high-level rifampicin resistance (Cambau et al., 2015), low-level resistance (Williams et al., 1998; Zaczek et al., 2009), as well it has been found in rifampicin susceptible strains (Somoskovi et al., 2006; Andres et al., 2014), therefore a non-consensual genotype associated to resistance important to be included and analyzed in the context of this study.

Regarding second-line drugs, the MDR strains MtbBR6 and MtbPT8 were additionally resistant to one second line-injectable drug, and MtbBR2 was resistant to ofloxacin. MtbBR6 and MtbPT8 harbored a mutation in *rrs*, A1401G, and in strain MtbBR2 was found the A90V mutation in *gyrA*. MtbPT9, MtbPT10, and MtbPT11 were classified as XDR. Strains MtbPT10 and MtbPT11 harbored the *rrs* A1401G. This is the most common mutation found in clinical isolates resistant to second-line aminoglycosides (Cambau et al., 2015; Domínguez et al., 2016). For MtbPT9, no mutation could be found in the *rrs* gene. The resistance to amikacin was assigned to a mutation in *eis* promoter and the resistance to capreomycin was attributed to the insertion of a GT base pair at position 755/756 of the *tlyA* gene. The mutation G-10A in the *eis* promoter region was previously showed to be associated with resistance to kanamycin (Zaunbrecher et al., 2009) and with low-level resistance to amikacin (Perdigão et al., 2013). The insertion of a GT base pair at position 755/766 of *tlyA* was previously showed to be associated with capreomycin resistance (Perdigão et al., 2013). Resistance to ofloxacin of these three strains was associated with common mutations in *gyrA* at codons 91 and 94 (Table 2; Cambau et al., 2015; Domínguez et al., 2016). MtbBR1 exhibited monoresistance to ofloxacin associated with a mutation in *gyrA*, D94N.

Summing up, we have selected a representative and diverse sample of clinical strains of *M. tuberculosis* from two geographical distinct areas harboring the most representative gene mutations in known drug targets, accounting for clinical resistance to the most important first- and second-line antituberculosis drugs. As per the most commonly accepted dogma about the mechanisms accounting for drug resistance in *M. tuberculosis*, the sole cause of the resistance levels noticed in these strains are expected to be derived exclusively from the diminished affinity of the mutated genes toward the respective antibiotic (Zhang and Yew, 2009; Domínguez et al., 2016).

### Quantification of the drug resistance levels in the presence of efflux inhibitors

Secondly, we quantified the drug resistance levels of the strains toward the antibiotics in study. For this purpose, the MICs of the antibiotics and efflux inhibitors were determined for each strain and the results are presented in Table 3. The MICs of the efflux inhibitors were found to be quite homogenous among the seven groups of strains. Conversely, the MICs of the antibiotics showed that for the same mutation different resistance levels were present. Therefore, we hypothesize that the different resistance levels presented for the same genotype are due to different levels of active efflux of the antibiotics. The existence of active efflux was evaluated indirectly through the reduction of the resistance levels of the antibiotics in the presence of the efflux inhibitors verapamil, thioridazine and chlorpromazine by qDST and MIC determination (Table 4).

**Table 3:** **MIC of antibiotics and efflux inhibitors for the *M. tuberculosis* strains studied**.

**Strain ID**	**MIC (μg/ml)**							
	Antibiotics^*^					**Efflux inhibitors**		
	**INH**	**RIF**	**AMK**	**CAP**	**OFX**	**VP**	**TZ**	**CPZ**
**SUSCEPTIBLE**								
H37Rv	0.1	1	1	2.5	1	256	15	30
MtbPT1	0.1	1	1	2.5	1	256	30	30
MtbPT2	0.1	1	1	2.5	1	256	15	30
**INH^R^**								
MtbPT3	10	1	1	2.5	1	256	15	30
MtbPT4	0.4	1	1	2.5	1	256	15	30
**RIF^R^**								
MtbPT5	0.1	256	1	2.5	1	256	15	30
MtbPT6	0.1	512	1	2.5	1	256	15	30
**OFX^R^**								
MtbBR1	0.1	1	1	2.5	32	128	15	30
**Poly-DR**								
MtbBR2	3	1	1	2.5	10	256	15	30
**MDR**								
MtbBR3	10	320	1	2.5	1	256	15	15
MtbBR4	10	640	1	2.5	1	256	15	15
MtbBR5	10	160	1	2.5	1	128	15	15
MtbPT7	3	320	1	2.5	1	256	15	30
MtbBR6	80	80	320	5	1	128	30	30
MtbPT8	20	320	40	25	1	256	15	30
**XDR**								
MtbPT9	20	80	4	25	10	128	15	30
MtbPT10	3	320	640	25	10	256	15	30
MtbPT11	3	320	640	50	10	256	15	30

* *The lowest concentration tested corresponded to the critical concentration for each antibiotic (see Section Materials and Methods for details). AMK, amikacin; CAP, capreomycin; CPZ, chlorpromazine; INH, isoniazid; MDR, multidrug resistant; OFX, ofloxacin; Poly-DR, poly-drug resistant; RIF, rifampicin; TZ, thioridazine; VP, verapamil; XDR, extensively drug resistant*.

**Table 4:** **Quantitative drug susceptibility testing for isoniazid, rifampicin, amikacin, and ofloxacin in the presence and absence of efflux inhibitors**.

**Antibiotic**		**Quantitative susceptibility testing (μg/ml)**															
		**Suscep**.	**INH**^**R**^		**RIF**^**R**^		**OFX^R^**	**Poly-DR**	**MDR**						**XDR**		
		**H37Rv**	**MtbPT3**	**MtbPT4**	**MtbPT5**	**MtbPT6**	**MtbBR1**	**MtbBR2**	**MtbBR3**	**MtbBR4**	**MtbBR5**	**MtbPT7**	**MtbBR6**	**MtbPT8**	**MtbPT9**	**MtbPT10**	**MtbPT11**
**INH**	**No EI**	0.1	10	0.4	–	–	–	10	10	10	10	3	80	20	20	3	3
	**+VP**	0.1	10	0.4	–	–	–	**3**	**3**	**3**	**3**	**1**	**3**	10	**1**	**1**	**1**
	**+TZ**	0.1	**3**	0.4	–	–	–	**3**	**3**	10	**3**	**1**	**3**	**3**	20	**1**	**3**
	**+CPZ**	0.1	**3**	0.4	–	–	–	**1**	10	10	**3**	**1**	**3**	**3**	**1**	**1**	**1**
**RIF**	**No EI**	1	–	–	256	512	–	1	320	640	160	320	80	320	80	320	320
	**+VP**	1	–	–	**32**	**32**	–	**<0.125**	**40**	**40**	**20**	**20**	**20**	**20**	**20**	**20**	**20**
	**+TZ**	1	–	–	256	**32**	–	**<0.125**	**80**	320	**40**	320	80	320	**20**	320	320
	**+CPZ**	1	–	–	**32**	**20**	–	**<0.125**	**80**	320	**40**	320	**20**	**20**	**4**	**20**	**20**
**AMK**	**No EI**	**1**	–	–	–	–	–	–	–	–	–	–	320	40	4	640	640
	**+VP**	1	–	–	–	–	–	–	–	–	–	–	320	**1**	4	640	640
	**+TZ**	1	–	–	–	–	–	–	–	–	–	–	320	**1**	4	640	640
	**+CPZ**	1	–	–	–	–	–	–	–	–	–	–	320	**1**	4	**40**	**40**
**CAP**	**No EI**	2.5	–	–	–	–	–	–	–	–	–	–	5	25	25	25	50
	**+VP**	2.5	–	–	–	–	–	–	–	–	–	–	5	25	25	**5**	**5**
	**+TZ**	2.5	–	–	–	–	–	–	–	–	–	–	**2.5**	25	25	**5**	25
	**+CPZ**	2.5	–	–	–	–	–	–	–	–	–	–	**2.5**	25	25	**5**	**5**
**OFX**	**No EI**	1	–	–	–	–	32	10	–	–	–	–	–	–	10	10	10
	**+VP**	1	–	–	–	–	16	**2**	–	–	–	–	–	–	10	10	10
	**+TZ**	1	–	–	–	–	16	**<1**	–	–	–	–	–	–	10	10	10
	**+CPZ**	1	–	–	–	–	16	**<1**	–	–	–	–	–	–	10	10	10

*The lowest concentration tested corresponds to the critical concentration for each antibiotic except for rifampicin testing for strain MtbBR2 (see Section Materials and Methods for details). Synergistic interactions are in bold; –, not done. AMK, amikacin; CAP, capreomycin; CPZ, chlorpromazine; EI, efflux inhibitor; INH, isoniazid; MDR, multidrug resistant; OFX, ofloxacin; RIF, rifampicin; TZ, thioridazine; VP, verapamil; XDR, extensively drug resistant*.

#### Resistance levels for isoniazid

Eleven out of 12 strains resistant to isoniazid presented high-level resistance (R ≥ 1 μg/ml). Efflux activity was detected in all except MtbPT4, the only strain that showed low-level resistance. The low-level resistance presented by this strain was attributed to the single C-15T mutation, which results in *inhA* overexpression and leads to titration of isoniazid and consequent mild increased resistance. For the remaining 11 strains, the resistance levels were reduced from high- to low-level (R < 1 μg/ml) in 4/11 with verapamil; 2/11 with thioridazine; and 5/11 with chlorpromazine. For the strains that do not reached low-level resistance, the MICs were reduced to 3 μg/ml. In respect to the efflux levels of the strains that harbored mutations in *katG*, the results showed that they have less efflux activity than those presenting mutations in *inhA*. Strains MtbPT7, MtbPT9, MtbPT10, and MtbPT11 have the C-15T mutation and additionally have a mutation in the *inhA* ORF contributing to their high-level resistance to isoniazid, as previously shown (Machado et al., 2013). The level of resistance of these strains was reduced with the efflux inhibitors to values always above that conferred by the single mutation in the *inhA* promoter (0.4 μg/ml), suggesting that the net resistance level conferred by *inhA* double mutations (promoter plus ORF) bears also a strong contribution from efflux. Noteworthy is that the strain MtbBR6 with a rare mutation in *katG* presents high-level resistance that could be reduced by all inhibitors tested. The difference between the levels of resistance caused by this mutation compared with those presented by the strains with the *katG* S315T mutation may be due to fitness cost. It is known that the mutation S315T has no fitness cost for the bacteria (Gagneux, 2009) being the most frequent and successful *katG* mutation accounting for isoniazid resistance worldwide (Seifert et al., 2015). Efflux activity is minimal in strains that bare this mutation since they don't need efflux to cope with the presence of isoniazid. These results confirm our previous findings that the high-levels of resistance associated with mutations in *inhA* have an increased component of efflux when compared with *M. tuberculosis* strains with high-level resistance associated with the *katG* S315T mutation (Machado et al., 2016).

#### Resistance levels for rifampicin

All the strains resistant to rifampicin presented high-level resistance (R ≥ 20 μg/ml). Efflux activity was detected in all with all the inhibitors although with different efficiencies (Table 4). The resistance levels toward rifampicin were significantly reduced in the presence of verapamil (11/11), thioridazine (4/11), and chlorpromazine (9/11). To assess the contribution of efflux activity to the borderline susceptibility toward rifampicin in MtbBR2, this strain was evaluated for susceptibility to rifampicin in the presence of efflux inhibitors. Notably, the borderline susceptibility was reversed with all inhibitors tested to concentrations well below the critical concentration (Table 4). We hypothesize that the *rpoB* D516Y mutation is associated with intermediate rifampicin susceptibility that has a strong association with efflux activity. For all other strains, the results demonstrated that efflux acts synergistically with mutations associated with rifampicin resistance although the *rpoB* S531L mutation is sufficient to produce high-levels of resistance to this antibiotic.

#### Resistance levels for amikacin and capreomycin

The *rrs* A1401G is associated with high-levels resistance to amikacin and capreomycin (Sirgel et al., 2011; Du et al., 2013). Strains MtbBR6, MtbPT8, MtbPT10, and MtbPT11 share the mutation A1401G. Efflux activity was detected in all except in MtbBR6 (Table 4). Amikacin resistance levels of MtbPT10 and MtbPT11 were reduced with chlorpromazine from 640 to 40 μg/ml, a 16-fold reduction. However, although significant, this reduction was not sufficient to reach amikacin low-level resistance. The low-level resistance to amikacin of MtbPT9 was due to a mutation in the *eis* promoter region and no reduction was obtained with any of the inhibitors tested. Amikacin is acetylated by Eis (Zaunbrecher et al., 2009). We hypothesize that the structural modification of the drug impairs substrate recognition and consequently its efflux by the efflux pumps but more studies are required to demonstrate this. Strain MtbPT8 showed the lowest level of resistance to amikacin (40 μg/ml) when compared with the other three amikacin resistant strains with the *rrs* A1401G mutation. In this strain, the resistance to amikacin was reversed with all the efflux inhibitors. This strain is heteroresistant to amikacin, i.e., the strain population is composed by an amikacin-susceptible subpopulation and an amikacin-resistant subpopulation. Heteroresistance was detected by reverse hybridization using the MTBDR*sl* assay. The detection of mixed genotypes (hybridization with both wild-type and resistant probes) in the same sample is dependent on the concentration of each individual genotype. Zhang et al. (2013) showed that the low-level of amikacin resistance presented by heteroresistant strains in mainly due to the presence of the drug susceptible population. This data indicates that the proportion of the amikacin-resistant subpopulation is reduced comparatively with the amikacin-susceptible subpopulation only conferring low-level resistance. Therefore, the resistant population during the acquired resistance pathway could be highly reduced in the presence of efflux inhibitors, further demonstrating the contribution of efflux to the establishment and stabilization of mutations in a *M. tuberculosis* population under drug selection (Machado et al., 2012; Schmalstieg et al., 2012).

Of the five strains resistant to capreomycin, four have the mutation *rrs* A1401G (including the heteroresistant one) and one presented a mutation in *tlyA*. With the exception of MtbBR6 all the others presented high-level resistance toward this antibiotic. The low-level resistance presented by MtbBR6 was reversed with thioridazine and chlorpromazine. Capreomycin resistance was reduced to low-level with verapamil (2/5), chlorpromazine (2/5), and thioridazine (1/5). Interesting, capreomycin resistance could not be reduced with any of the inhibitors tested in the heteroresistant strain (MtbPT8) for which amikacin resistance was reversed. Since cross-resistance between amikacin and capreomycin is due to the mutation *rrs* A1401G, this unexpected result may be explained by the existence of an unknown mechanism associated with capreomycin resistance in this strain. We search for mutations in *tlyA* that could co-exist with the *rrs* mutation but none was found and this subject will be explored in future work. It was not possible to reduce capreomycin resistance in MtbPT9 which is associated with an insertion mutation in *tlyA*. Capreomycin is methylated by TlyA and the loss of methyltransferase activity due to the presence of mutations in *tlyA* reduces susceptibility to this drug (Maus et al., 2005). Similar to that observed for Eis, we can speculate that the alteration of an essential pathway necessary for the binding of the drug to the target will hamper the substrate recognition and consequently its efflux by the mycobacterial efflux pumps.

#### Resistance levels for ofloxacin

Resistance to fluoroquinolones involve mainly mutations in *gyrA* and the most common are at positions 90, 91, and 94 which are associated with high-level resistance (Cambau et al., 2015). All the strains resistant to ofloxacin presented high-level resistance (Table 4). Resistance to ofloxacin of strains MtbPT9 to MtbPT11 could not be reduced with any of the inhibitors tested. On these strains, resistance could be solely attributed to the mutations S91P and D94A on *gyrA*. For MtbBR1 only basal efflux activity could be detected (Table 4). Contrary, ofloxacin resistance of strain MtbBR2 was reversed by thioridazine and chlorpromazine to levels below the critical concentration and reduced from 10 to 2 μg/ml with verapamil (Table 4). This strain harbored the *gyrA* A90V mutation whose role in ofloxacin resistance is controversial. The *gyrA* A90V mutation was recently associated with borderline levels of resistance toward ofloxacin, i.e., the level of resistance can be close or below the critical concentration of 1 μg/ml (Niward et al., 2016). In contrast, Farhat et al. (2016) reported that strains with the *gyrA* A90V mutation have a comparable effect on ofloxacin MIC as the mutation D94A/G (1 μg/ml>MIC<10 μg/ml). Other studies associate this mutation with ofloxacin resistance above 2 μg/ml (Poissy et al., 2010; Cambau et al., 2015). Our results showed that the A90V mutation can confer resistance up to 2 μg/ml and that the remaining resistance presented has an efflux component that can be reduced with an efflux inhibitor. This mutation have also been found in strains harboring both mutated and wild-type sequences, i.e., fluoroquinolone heteroresistant strains, or in samples harboring two different strains (van den Boogaard et al., 2011). The discrepancies noted between these studies together with the evidence that efflux in linked to this mutation may indicate that the mutation *gyrA* A90V is associated with intermediate resistance to ofloxacin in strong association with fluoroquinolone efflux that increases the levels of resistance, nevertheless, more studies are needed to support this hypothesis. All other *gyrA* mutations detected were sufficient to produce stable ofloxacin resistance demonstrating that efflux has little contribution toward ofloxacin resistance when these mutations are present.

### Determination of the intrinsic efflux capacity of the *M. tuberculosis* strains

**Figure 1:**
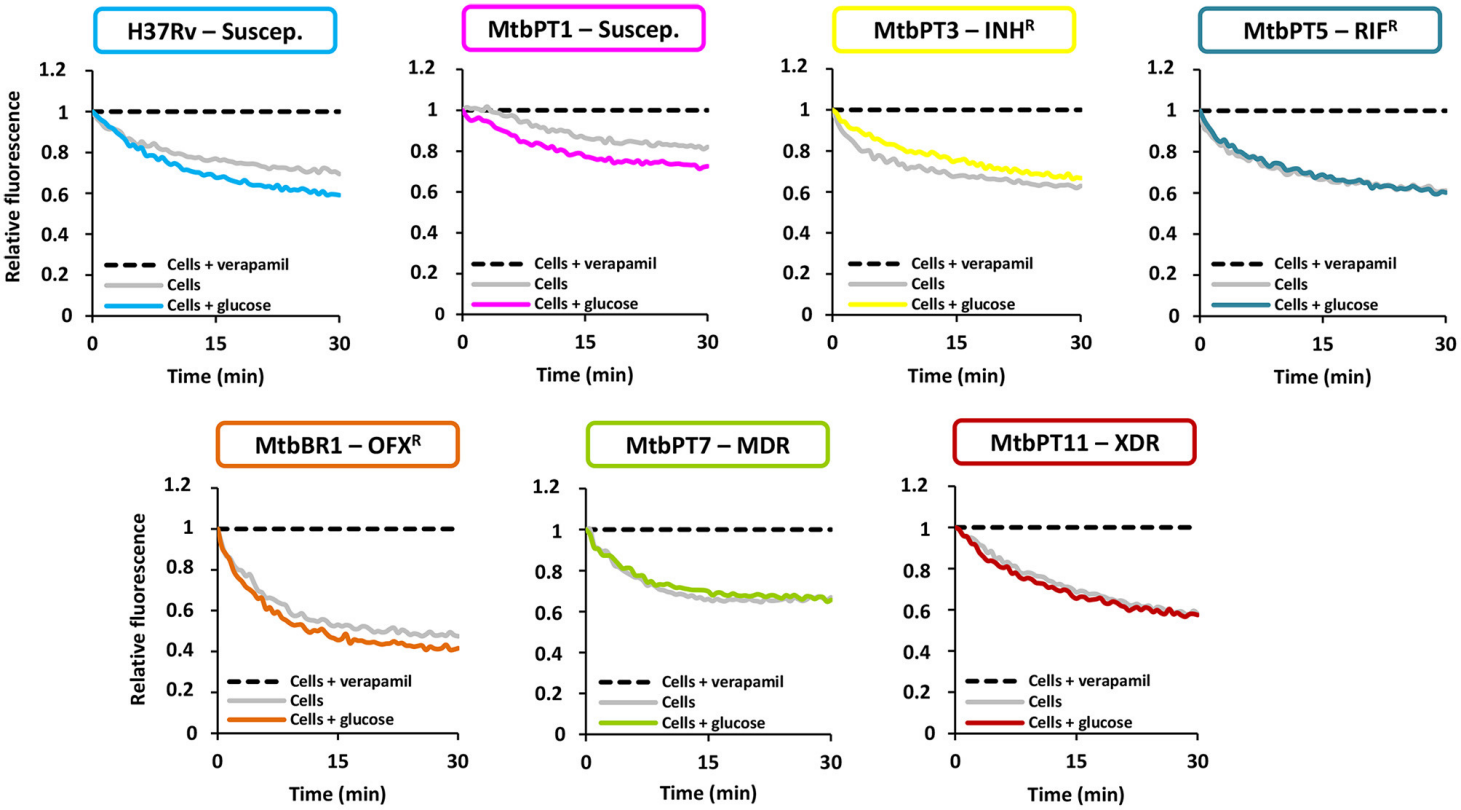
**Assessment of efflux activity on the *M. tuberculosis* strains in study**. Ethidium bromide was used at the equilibrium concentration for each strain as follows: 0.25 μg/ml for H37Rv, MtbPT1, MtbPT5, MtbPT7, and MtbPT11; 0.5 μg/ml, MtbPT3; and 1 μg/ml, MtbBR1; verapamil was tested at half MIC.

**Figure 2:**
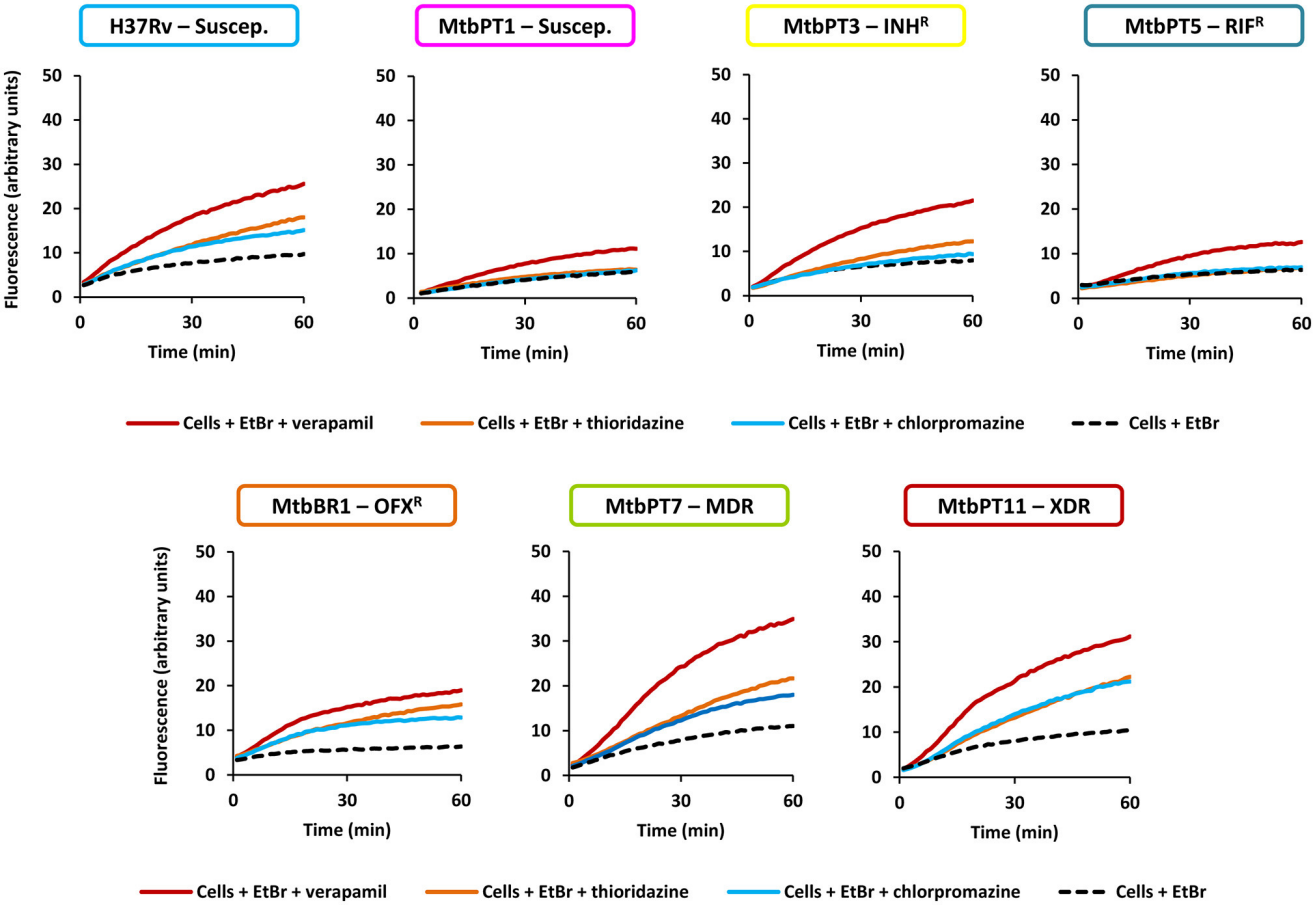
**Evaluation of the effect of efflux inhibitors on the accumulation of ethidium bromide on the *M. tuberculosis* strains in study**. Ethidium bromide (EtBr) was used at the equilibrium concentration for each strain as follows: 0.25 μg/ml for H37Rv, MtbPT1, MtbPT5, MtbPT7, and MtbPT11; 0.5 μg/ml, MtbPT3; and 1 μg/ml, MtbBR1; each efflux inhibitor was tested at half MIC.

Thirdly, to confirm the existence of active efflux systems on these strains we have evaluated their ability to efflux ethidium bromide, a fluorescent efflux substrate, by real-time fluorometry (Paixão et al., 2009; Viveiros et al., 2010; Machado et al., 2012). To perform these assays, one representative of each drug resistant group was selected: MtbPT3, monoresistant to isoniazid; MtbPT5, monoresistant to rifampicin; MtbBR1, monoresistant to ofloxacin; MtbPT7, multidrug resistant, and MtbPT11, extensively drug resistant (Figure 1). *M. tuberculosis* H37Rv and MtbPT1 were included as representatives of drug susceptible strains. The strains were incubated with ethidium bromide at the equilibrium concentration determined for each strain and verapamil at half MIC (see Table 3 for MIC values) for 1 h. After this period, verapamil was washed out from the solution, the cells were assayed with glucose, as energy source, and without glucose, and their efflux capacity was measured by fluorometry (see Section Materials and Methods for details). As can be observed in Figure 1, efflux of ethidium bromide was detected in all these strains (colored curve), although with different efficiencies, which was inhibited in presence of verapamil at half MIC (black dashed line). It was also noted that the efflux of ethidium bromide is not affected by the presence of glucose (compare gray curve with the colored curve on each graph) which shows that the external energization of the cells is not necessary to guarantee an optimal efflux activity in *M. tuberculosis*, a common requirement for efficient efflux in Gram-negative bacteria (Paixão et al., 2009; Viveiros et al., 2010). The assays performed with the susceptible strains further confirmed that efflux activity in *M. tuberculosis* is an intrinsic characteristic of susceptible and drug resistance strains (Machado et al., 2012). The different degrees of efflux activity observed, e.g., MtbBR1 presented increased efflux activity and MtbPT1 presented reduced efflux activity compared with the other strains, may be related to the different environmental conditions to which these strains were subject in the clinical setting. To further confirm that the efflux activity of these strains could be inhibited in the presence of the efflux inhibitors, we performed ethidium bromide accumulation assays in presence of verapamil, thioridazine, and chlorpromazine (Figure 2) and determined the RFF index (Table 5). The RFF index is a measure of how efficient is the inhibitor in promoting intracellular accumulation of ethidium bromide. The results showed that the three inhibitors were capable to increase the ethidium bromide accumulation in all the tested strains (Table 5). The most efficient efflux inhibitor in these assays was verapamil (6/7), followed by thioridazine (2/7), and chlorpromazine (1/7). The results demonstrated that these compounds are able to promote intracellular accumulation of ethidium bromide on *M. tuberculosis* susceptible, monoresistant and MDR/XDR strains, clearly putting in evidence that active efflux is inhibited by these compounds.

**Table 5:** **RFF based on the accumulation of ethidium bromide for the *M. tuberculosis* strains in the presence of efflux inhibitors**.

**Strain**	**RFF of the inhibitors**		
	**Verapamil**	**Thioridazine**	**Chlorpromazine**
H37Rv	1.69 ± 0.09^***^	0.82 ± 0.15^*^	0.81 ± 0.03^**^
MtbPT1	0.86 ± 0.03^**^	0.18 ± 0.06^*^	0.13 ± 0.02^*^
MtbPT3	1.68 ± 0.24^**^	0.78 ± 0.02^*^	0.45 ± 0.02^*^
MtbPT5	1.17 ± 0.08^**^	0.24 ± 0.09	0.26 ± 0.06
MtbBR1	**1.76 ± 0.21**	**1.39 ± 0.32**	0.88 ± 0.13
MtbPT7	2.17 ± 0.01^*^	0.98 ± 0.02^*^	0.67 ± 0.04^*^
MtbPT11	2.00 ± 0.21^**^	1.14 ± 0.11^*^	1.04 ± 0.03^**^

Ethidium bromide was used at the equilibrium concentration for each strain as follows: 0.25 μg/ml, H37Rv, MtbPT1, MtbPT5, MtbPT7, and MtbPT11; 0.5 μg/ml, MtbPT3; and 1 μg/ml, MtbBR1; the inhibitors were tested at half MIC. The effect of the inhibitors on the accumulation of ethidium bromide was interpreted as follows: RFF values above zero indicated that the cells accumulate more ethidium bromide under the condition used than those of the control (non-treated cells). Values in boldface (above 1) indicated enhanced accumulation of ethidium bromide in the presence of the efflux inhibitors. Each assay was performed in triplicate and the results presented correspond to the average of three independent assays plus standard deviation (±SD). The results were considered significant when

* P < 0.05; and highly significant when

** P < 0.01 and

*** *P < 0.001*.

### Effect of antibiotics on efflux pump gene expression

**Figure 3:**
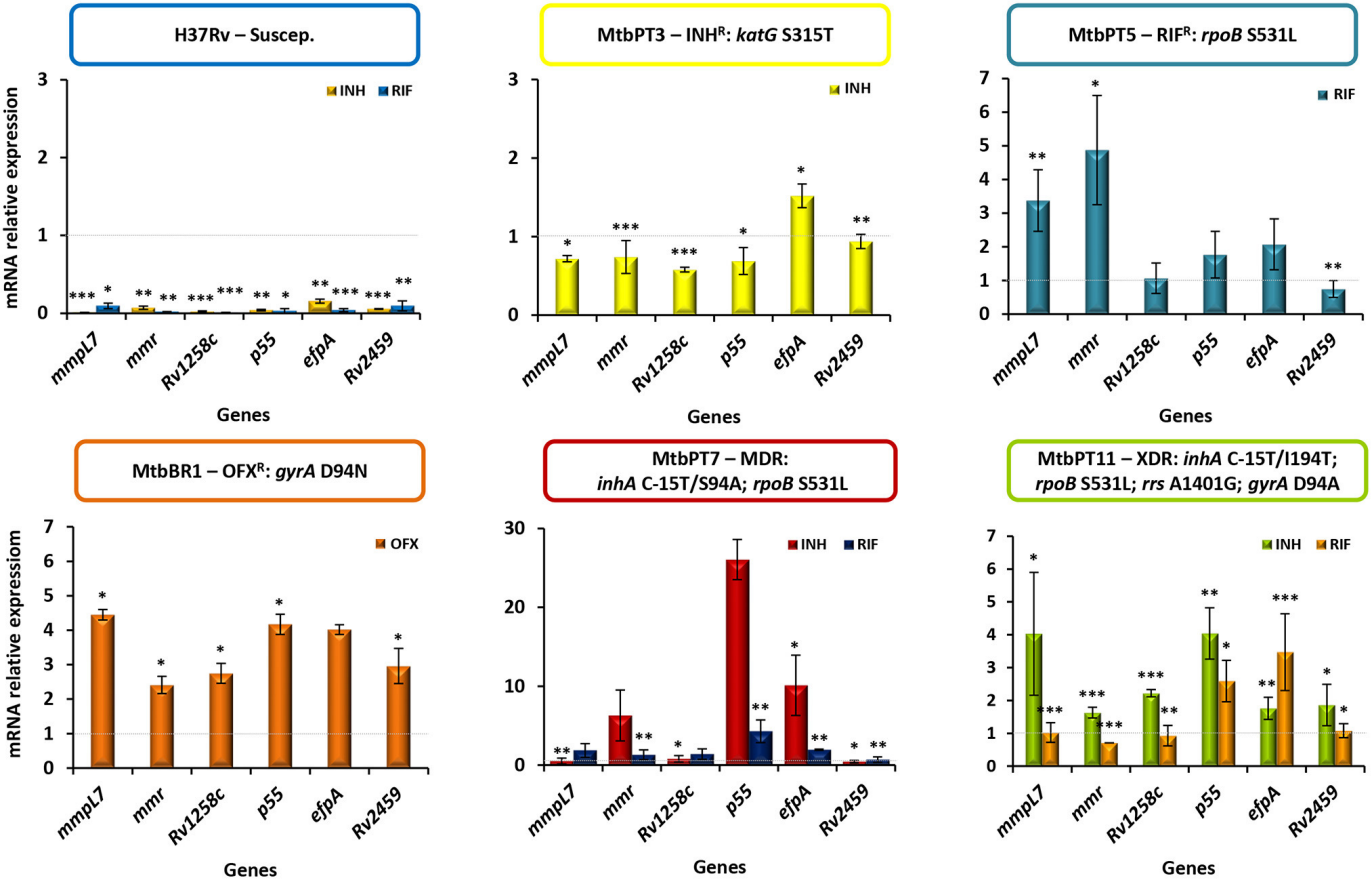
**Quantification of the relative mRNA expression levels of a panel of efflux pump genes**. Strains were grown in MGIT tubes for the MGIT 960 system in the presence of half MIC of each antibiotic as follows: MtbPT3 was exposed to isoniazid; MtbPT5 was exposed to rifampicin; MtbBR1 was exposed to ofloxacin; H37Rv, MtbPT7, and MtbPT11 were exposed to isoniazid or rifampicin. The relative expression of the efflux pump genes was assessed by comparison of the relative quantity of the respective mRNA in the presence of an antibiotic to the non-exposed strain. A level of relative expression equal to 1 indicates that the expression level was identical to the strain that was being compared (gray dashed line in the graphs). Genes showing expression levels above 1 were considered to be expressed; genes showing expression levels above two were considered to be significant overexpressed. The results were considered significant when ^*^P < 0.05; and highly significant when ^**^P < 0.01 and ^***^P < 0.001.

To further corroborate these findings we analyzed the transcriptional profile of putative efflux pumps described in the literature as being associated with antibiotic resistance phenotypes of *M. tuberculosis* strains (Viveiros et al., 2012; Black et al., 2014; da Silva et al., 2016). RT-qPCR was done for six genes previously shown to be overexpressed in response to antibiotic exposure (Machado et al., 2012, 2016). The results showed the occurrence of significant changes in the expression levels in all strains upon exposure to the antibiotics except in the susceptible H37Rv strain (Figure 3). Concerning the monoresistant strains, in MtbPT3 exposed to isoniazid only *efpA* was found overexpressed. In strain MtbPT5 exposed to rifampicin, from the six genes tested, four were found overexpressed namely *mmpL7, mmr, p55*, and *efpA*. The ofloxacin resistant strain MtbBR1 when exposed to ofloxacin showed significant overexpression all the genes tested (≥two-fold expression). The MDR and XDR strains were exposed to rifampicin or isoniazid and similar patterns were observed. Three to six genes were overexpressed upon exposure to isoniazid or rifampicin. P55 showed the highest level of expression on both strains and for both antibiotics. Overall, EfpA was overexpressed in all strains independently of the antibiotic to which it was exposed. Contrary, Rv2459 was found overexpressed only in the ofloxacin monoresistant strain exposed to ofloxacin. For the remaining efflux pumps analyzed we could not associate an antibiotic to the overexpression of a particular efflux pump. We have observed a general pattern of efflux pump gene expression upon the exposure to the antibiotics.

### *M. tuberculosis* growth kinetics in the presence of antibiotics and efflux inhibitors

If efflux really contributes to the overall drug resistance level seen above, the delay in the growth of each drug resistant strain, due to the stress imposed by the combination of an antibiotic plus an efflux inhibitor at subinhibitory concentrations, will render them more susceptible to the effect of the antibiotic. To test this hypothesis, we performed qDST for selected *M. tuberculosis* strains and investigated the TTD of growth in the presence and absence of the efflux inhibitors to attain clinical correlation (Machado et al., 2016).

**Figure 4:**
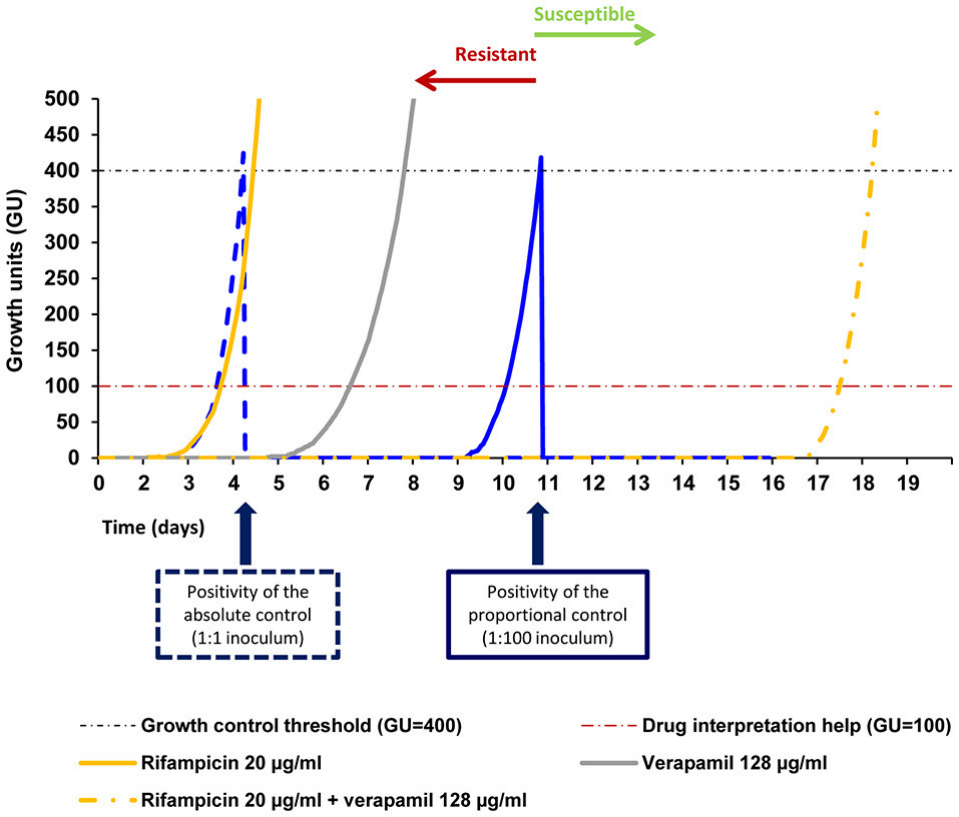
**Quantitative drug susceptibility testing of rifampicin for the strain MtbPT11, in the presence or absence of verapamil**. Quantitative drug susceptibility testing of isoniazid was conducted using the BACTEC 960 system and the Epicenter V5.80A software equipped with the TB eXIST module. In the Figure is presented the result of the testing for the strain MtbPT11 at 20 μg/ml of rifampicin alone (full orange curve) and rifampicin at 20 μg/ml in the presence of verapamil at half MIC (dashed orange curve). Verapamil alone does not inhibit the growth of the strain (gray curve). Absolute control is given by the dotted blue curve (undiluted strain 1:1) and the proportional control is given by the continuous blue curve (strain diluted 1:100). At the time of growth of the proportional growth control (GU = 400—black dashed line), the comparison between this tube and the tubes containing the drugs(s) was performed. If the GU of the tubes containing the drug were >100 (red dashed line), they were considered to be resistant to that concentration. If the GU of the tube containing the drug was <100 they were considered susceptible.

**Figure 5:**
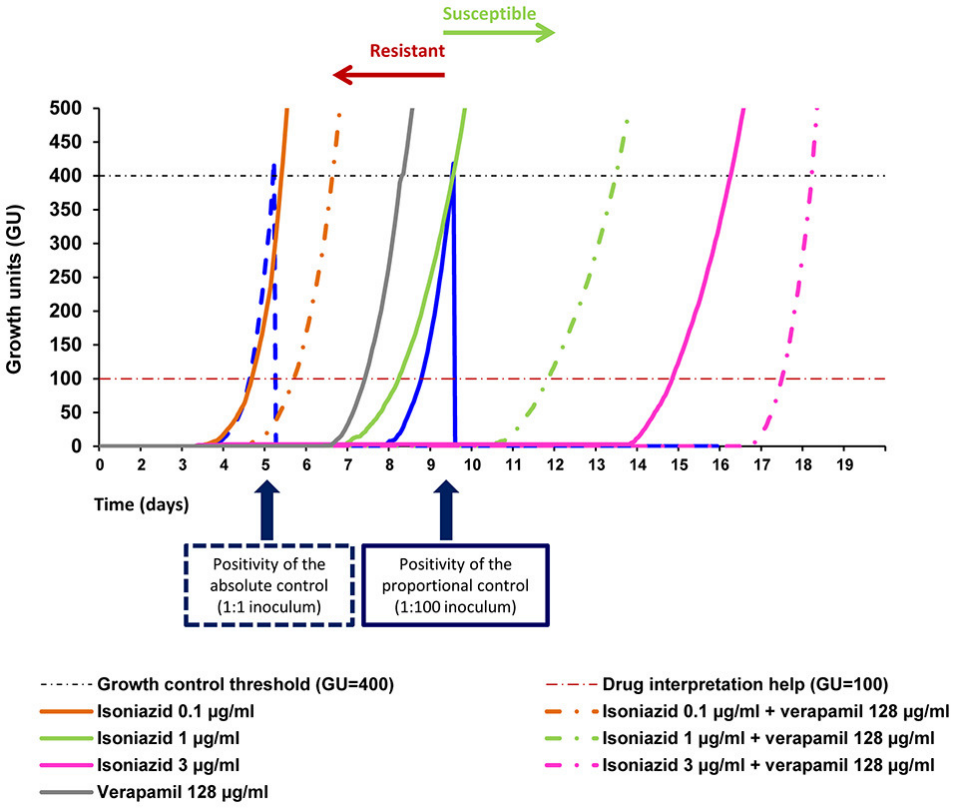
**Quantitative drug susceptibility testing of isoniazid for the strain MtbPT11, in the presence or absence of verapamil**. Quantitative drug susceptibility testing of isoniazid was conducted using the BACTEC 960 system and the Epicenter V5.80A software equipped with the TB eXIST module. Absolute control is given by the dotted blue curve (undiluted strain 1:1) and the proportional control is given by the continuous blue curve (strain diluted 1:100). The orange, yellow, pink, and green full curves correspond to the growth curves of the strain in the presence of only isoniazid; the respective colored dashed curves corresponded to the growth curves of the strain in the presence of isoniazid and verapamil; Verapamil alone does not inhibit the growth of the strain (gray curve). At the time of growth of the proportional growth control (GU = 400—black dashed line), the comparison between this tube and the tubes containing the drugs(s) was performed. If the GU of the tubes containing the drug were >100 (red dashed line), they were considered to be resistant to that concentration. If the GU of the tube containing the drug was <100 they were considered susceptible. Isoniazid was tested at 0.1, 1, 3, and 10 μg/ml with and without half MIC of verapamil.

The results showed a delay between the TTD of the tube containing the antibiotic plus an efflux inhibitor and the tube containing only the antibiotic ranging from 1 to 63 days depending on the antibiotic concentration and the drug combination (Supplementary Tables 2–4). Rifampicin at 1 μg/ml in combination with the efflux inhibitors showed a delay in the TTD of strain MtbPT11 of 48 h in the presence of thioridazine, corresponding to a potentiation of rifampicin activity of 63.23% when compared with rifampicin only. Similarly, in the presence of verapamil or chlorpromazine, the delay was 72 h corresponding to a potentiation of rifampicin activity of 95.81 and 96.77%, respectively. Noteworthy, this strain became susceptible to rifampicin at 20 μg/ml in the presence of verapamil (Figure 4) and chlorpromazine, with a delay in the TTD of 386 and 195 h, respectively (Supplementary Table 2). In Figure 5 is presented the qDST of isoniazid for the same strain and it can be observed the increasing of the TTD at each concentration of isoniazid in the presence of half MIC of verapamil. These results showed that the antibiotic activity is clearly potentiated by the efflux inhibitors as demonstrated by the delay on the TTD of growth although, as seen previously, they do not reach susceptibility levels due to the antibiotic-target mutations they carry.

## Discussion

Whilst drug resistance in *M. tuberculosis* has long been associated with the development of mutations in the genes that code for the drug targets, efflux pump activity was only recently recognized to play a significant role in the development of drug resistant phenotypes in *M. tuberculosis*. Several recent studies have demonstrated the importance of the overexpression of efflux pump genes in MDR and XDR *M*. *tuberculosis* clinical strains (Calgin et al., 2013; Coelho et al., 2015; Li et al., 2015a; Yamchi et al., 2015; Kanji et al., 2016; Machado et al., 2016; Oh et al., 2017), in rifampicin monoresistant strains (Li et al., 2015b), or in the H37Rv susceptible strain after exposure to drugs (Garima et al., 2015; Caleffi-Ferracioli et al., 2016). Nevertheless, most of these studies are based on the simple assessment and evaluation of the levels of expression of *M. tuberculosis* efflux pump genes, and few have determined the effect of efflux inhibitors on the MICs of the antituberculosis drugs and have quantified the activity of the overexpressed efflux systems. Therefore, further studies are needed to explore the contribution of the overactivity of efflux pumps to the resistance levels in *M. tuberculosis* strains.

In a previous work, we presented a model on how multidrug resistance develops in tuberculosis patients, the role of efflux pumps on the development of isoniazid resistance once exposed to the critical concentration of this antituberculosis drug and how these events determine the basis of acquired multidrug resistance (Machado et al., 2012). From that work two questions were raised: (i) what is the role played by the efflux systems in *M. tuberculosis* strains that already carry mutations in the drug target genes? and (ii) do they respond in the same way to the drug pressure despite the presence of a mutation? In this work, we selected a panel of *M. tuberculosis* strains presenting different phenotypes and genotypes to study the relationship between the effects of efflux inhibitors on the levels of resistance of the main antituberculosis drugs, the overexpression of efflux pumps upon drug pressure, and the presence of active efflux systems. This study was designed and executed to provide a simple and comprehensive demonstration that drug resistance in *M. tuberculosis* is a combination between efflux and the presence of a mutation in the drug target gene, a controversial subject in debate for many years (Jarlier and Nikaido, 1994; Louw et al., 2009; da Silva et al., 2011, 2016; Machado et al., 2012; Schmalstieg et al., 2012; Viveiros et al., 2012).

First we analyzed the effect of verapamil, thioridazine and chlorpromazine, known inhibitors of *M. tuberculosis* efflux pumps, on the resistance levels of first- and second-line drugs against a panel of monoresistant, MDR and XDR *M. tuberculosis* strains. The results obtained demonstrated that the resistance levels of the *M. tuberculosis* clinical strains toward the first- and second-line antibiotics studied could be reduced by an efflux inhibitor, independently of the genotype of the strains, demonstrating that the efflux pump inhibitors could be useful in combination with the standard antituberculosis therapy. These results showed that the drug resistant level observed on these strains is a combination between the efflux activity and the mutation in a drug resistance target. With the concentrations of the efflux inhibitors that we tested (half MIC), we can only aim to reduce the efflux component but the resistance component due to the mutation remains untouched. It is not plausible that a compound, whose main function is the inhibition of efflux activity, could reverse the antibiotic resistance in the presence of a mutation associated with high-level resistance. Since all these strains presented mutations associated with high-level drug resistance, these antibiotics are useless in these cases. Although the degree of synergism does not reach full susceptibility, the reduction of the MIC value of the antibiotics to values close or within the serum concentration can improve therapy outcomes demonstrating the possible usefulness of the efflux inhibitors as helper-compounds of antibiotic activity in tuberculosis treatment (Böttger, 2011; Machado et al., 2012; Viveiros et al., 2012; da Silva et al., 2016). Furthermore, it is described that the antibiotics can penetrate and concentrate within macrophages (Prokesch and Hand, 1982) increasing, by this manner, their effective concentrations.

We also demonstrated that the efflux inhibitors verapamil, thioridazine, and chlorpromazine are able to promote the intracellular accumulation of ethidium bromide, a broad range substrate for efflux pumps (Blair and Piddock, 2016), on the *M. tuberculosis* strains, clearly putting in evidence that active efflux is inhibited by these compounds. At the present, we do not know the exact mechanism by which these compounds inhibit the efflux activity in *M. tuberculosis*. It has been hypothesized in several studies that these efflux inhibitors affect *M. tuberculosis* membrane energetics and that they inhibit efflux by inhibiting the energy required for the pumps function (Black et al., 2014; Pule et al., 2016; da Silva et al., 2016). Due to the paucity of new antituberculosis drugs in the tuberculosis drug discovery pipeline and the increasing of drug resistant tuberculosis there is an urgent need for the development of new drugs and the implementation of new therapeutic strategies. The use of compounds that have the ability to inhibit mycobacterial efflux pumps promoting the retention of the co-administered antibiotics that are subject to efflux will obviously improve the efficacy and will extend the clinical utility of the existing antibiotics. In this sense, in the last years, various compounds have been developed and described as putative efflux pump inhibitors, e.g., verapamil analogs were shown to be effective antituberculosis inhibitors being able to reduce the level of resistance to rifampicin by binding to the efflux pump Rv1258c (Singh et al., 2014); thioridazine derivatives have demonstrated to potentiate the antituberculosis drug activity against *M. tuberculosis in vitro* and *ex vivo* (Pieroni et al., 2015), and hybrid efflux pump inhibitors, combining the activities of verapamil and thioridazine, were also shown to possess *in vitro* antituberculosis activity and intracellular activity in macrophages (Kumar et al., 2016).

Next, we analyzed the expression levels of five putative efflux pumps upon exposure to the antibiotics in the H37Rv susceptible strain, and in the monoresistant, MDR and XDR *M. tuberculosis* clinical isolates. The efflux pump genes *mmr, mmpL7, Rv1258c, p55*, and *efpA* were shown to be overexpressed in presence of antibiotics demonstrating the contribution of these efflux pumps to the resistance phenotype of the strains studied, with the exception of the H37Rv strain. We have noticed a general overexpression of almost all efflux genes studied upon exposure to the antibiotics in the drug resistant strains independently on the genotype of the strains. Indeed, when we look to the substrate specificity of each of the *M. tuberculosis* efflux pumps described in the literature we cannot find a correlation between a given substrate and a specific efflux pump (Supplementary Table 1). These results indicated that *M. tuberculosis* efflux pumps are promiscuous in their activity as we cannot associate the extrusion of drugs to a specific gene. Moreover, the RT-qPCR data combined with the real-time fluorometry results showed that the drug resistant clinical strains are more prompt to respond, via an efflux-mediated response, to the antibiotics, whereas the susceptible H37Rv strain shows a less prompt detoxifying response to the drugs to which it was exposed. Altogether, the results obtained demonstrate that *M. tuberculosis* clinical strains are primed to efflux noxious compounds and showed that efflux pump induction is involved in the antibiotic resistance pattern of the strains in study, reinforcing the validity of the hypotheses that efflux contributes to the resistance level of these strains, contributing also to their MDR phenotype.

We also showed that the TTD of *M. tuberculosis* growth in the MGIT 960 culture system in combination with qDST is a valuable methodology to evaluate *M. tuberculosis* drug response and to characterize the potency of drug combinations for personalized treatment, especially for MDR/XDR-TB patient regimens. Since the TTD and the inoculum concentration are inversely correlated (Diacon et al., 2010; Bowness et al., 2015), this synergistic activity can be highly beneficial during tuberculosis therapy, either in drug susceptible or drug-resistant strains, allowing the antibiotic, combined with the host's immune system, to tackle in the most effective way the infecting strain. This method allowed the monitoring, on a real-time basis, of bacterial growth/survival vs. growth inhibition by the comparison between the TTD of growth of a given strain in the presence and absence of an efflux inhibitor. The methodology used proved to be useful to study the contribution of the efflux mechanisms to *M. tuberculosis* drug resistance and simultaneously evaluate the efficacy of an efflux inhibitor to decrease the level of resistance of a strain to a given drug in the perspective of the development of new therapeutic strategies for susceptible and drug resistant tuberculosis, incorporating efflux inhibitors in the control and prevention of drug resistance during therapy.

In conclusion, this study allowed us to show that the main mechanisms associated with drug resistance in *M. tuberculosis* correlates mutations in target genes with increased efflux and that compounds that inhibit efflux activity can significantly reduce the phenotypic level of such resistance. The level of drug resistance in *M. tuberculosis* is a combination between the presence of a mutation in the drug target genes and a general stress response to the presence of noxious compounds that regulates the intracellular level of a drug. The data obtained in the presented study corroborated our previous findings (Machado et al., 2012; Coelho et al., 2015) now tested on a larger and diverse panel of *M. tuberculosis* clinical strains. The demonstration that the efflux activity modulates the levels of antibiotic resistance by complementing the resistance due to target-gene mutations, is a very relevant finding in the context of the ongoing discussion on the ability and clinical reliability of sole molecular based detection of the target-gene mutations as the future routine DST for *M. tuberculosis* (Böttger, 2011; Domínguez et al., 2016; Pankhurst et al., 2016; Schön et al., 2016). The use of efflux inhibitors as adjuvants of the antituberculosis therapy may be a promise for the development of new and shorter therapeutic strategies as they may potentiate the activity of the current antituberculosis drugs shortening the recommended 6 month treatment for cure, they can increase the activity of drugs that are no longer used due to the emergence of resistance, and they may also be used to protect the activity and usefulness of the new antituberculosis drugs from the development of drug resistance.

## Author contributions

DM and MV conceived and designed the study. DM, TC, JP, and CP performed the experiments. DM, TC, JP, IC, IP, RM, DR, AV, MR, PS, MV analyzed the data. IP, IC, and MV contributed with reagents/materials. DM and MV wrote the manuscript. All authors reviewed and approved the final version of the manuscript.

## Funding

This work was partially supported by projects PTDC/BIA-MIC/121859/2010 and UID/Multi/04413/2013 from Fundação para a Ciência e a Tecnologia (FCT), Portugal, and project “Ciência sem Fronteiras/Professor Visitante Especial” (Ref. 88881.064961/2014-01) from CAPES/MEC/Brazil.

### Conflict of interest statement

The authors declare that the research was conducted in the absence of any commercial or financial relationships that could be construed as a potential conflict of interest.
