# Supplementary material for: Interplay between Mutations and Efflux in Drug Resistant Clinical Isolates of Mycobacterium tuberculosis
Source: Front Microbiol. 2017 Apr 27;8:711. doi: 10.3389/fmicb.2017.00711 (PMC5406451; doi:10.3389/fmicb.2017.00711)
Supplement: Supplementary file 1 [file Table1.pdf]

Supplementary Material

Interplay between mutations and efflux in drug resistant *Mycobacterium tuberculosis* clinical isolates

Diana Machado[1§], Tatiane Coelho[2,3§], João Perdigão[4], Catarina Pereira[4], Isabel Couto[1], Isabel Portugal[4], Raquel Maschmann[2,5], Daniela Ramos[3], Andrea von Groll[3], Maria Lúcia Rossetti[5,6], Pedro A. Silva[2,3]† and Miguel Viveiros[1]†*

*** Correspondence:** Miguel Viveiros: mviveiros@ihmt.unl.pt

1 Supplementary Table

Supplementary Table 1. Putative efflux transporters described in *M. tuberculosis* and their substrates.

Transporter	Gene	Function	Substrates	Family	Energy source	Reference
Rv0194	*Rv0194*	Probable transmembrane multidrug efflux pump	AMP; CHL; ERY; EtBr; NOV; STR; TET; VAN	ABC	ATP	12
PstB	*pstB* (*Rv0933*)	Phosphate-transport ATP-binding protein	EMB; INH; FQs; RIF	ABC	ATP	5;8;14;39
Rv1218c–Rv1217c	*Rv1218c-Rv1217c*	Probable tetronasin-transport integral membrane protein	BL; BP; BSP; INH; NOV; PA; PD; PR; RIF	ABC	ATP	4;16:43
Rv1458c-Rv1457c-Rv1456c	*Rv1458c-Rv1457c-Rv1456c*	Probable unidentified antibiotic-transport integral membrane	EMB; INH; RIF; STR	ABC	ATP	22
Rv1747	*Rv1747*	Probable conserved transmembrane ATP-binding protein	EMB; EtBr; INH	ABC	ATP	7;41
BacA	*bacA* (*Rv1819c*)	Probable drug-transport transmembrane ATP-binding protein	AP; INH; RIF	ABC	ATP	17;20;25;27

Rv2688c-Rv2687c-Rv2686c	*Rv2688c-Rv2687c-Rv2686c*	Antibiotic-transport ATP-binding protein	FQs	ABC	ATP	7;33
DrrAB	*Rv2936-Rv2937*	Daunorubicin-dim-transport ATP-binding proteins	BCECF; CHL; DAU; DOX; EMB; ERY; EtBr; NOR; PUR; RIF; STR; TET	ABC	ATP	7;9;27;32
**Mmr**	***mmr* (*Rv3065*)**	**Multidrug-transport integral membrane protein**	**Dyes; ERY; INH; PY; TPP**	**SMR**	**PMF**	**3;15;30;38**
Rv1634	*Rv1634*	Possible drug efflux membrane protein	FQs; SKI	MFS	PMF	14;23
Rv0849	*Rv0849*	Probable conserved integral membrane transport protein	BL; INH; RIF	MFS	PMF	3
EmrB	*emrB* (*Rv0783c*)	Multidrug resistance integral membrane efflux protein	Several drugs	MFS	PMF	14
**EfpA**	***efpA* (*Rv2846c*)**	**Possible integral membrane efflux protein**	**ACR; EtBr; FQs; INH; RIF**	**MFS**	**PMF**	**19;27;30**
**P55**	***p55* (*Rv1410c*)**	**Aminoglycosides/tetracycline-transport integral membrane protein**	**AGs; CFZ; INH; RIF; TET**	**MFS**	**PMF**	**6;11;25;35**
**Rv1258c**	***Rv1258c (tap-like)***	**Probable conserved integral membrane transport protein**	**EMB; ERY; EtBr; FQs; INH; RIF; SPE; TET**	**MFS**	**PMF**	**1;25;30;37;40**
Stp	*stp* (*Rv2333c*)	Integral membrane drug efflux protein Stp	RIF; SPE;TET	MFS	PMF	36
Rv1877	*Rv1877*	Conserved membrane protein	ERY; KAN; TET	MFS	PMF	14
**Rv2459**	***Rv2459 (jefA)***	**Conserved integral membrane transport protein**	**EMB; EtBr; INH**	**MFS**	**PMF**	**14;21;30**
MmpL3	*mmpL3* (*Rv0206c*)	Probable conserved transmembrane transport protein	SQ109; BM212; AU; IA	RND	PMF	26;28;42
MmpL4	*mmpL4* (*Rv0450c*)	Probable conserved transmembrane transport protein	CMB; MB; RIF	RND	PMF	13;44
MmpL5	*mmpL5* (*Rv0676c*)	Probable conserved transmembrane transport protein	AZ; BDQ; CFZ; TET	RND	PMF	24;31
**MmpL7**	***mmpL7* (*Rv2942*)**	**Probable conserved transmembrane transport protein**	**INH**	**RND**	**PMF**	**18;30;34**
MmpL8	*mmpL8* (*Rv3823c*)	Probable conserved transmembrane transport protein	SQ109	RND	PMF	28
MmpL10	*mmpL10* (*Rv1183*)	Probable conserved transmembrane transport protein	SQ109	RND	PMF	28

IniBAC	*iniB* (*Rv0341*)<br>*iniA* (*Rv0342*)<br>*iniC* (*Rv0343*)	Isoniazid- inducible protein IniB<br>Isoniazid- inducible protein IniA<br>Isoniazid- inducible protein IniC	ETB; INH	Membrane protein	-	2;10

ABC, ATP-binding cassette; ACR, acriflavine; AGs, aminoglycosides; AMP, ampicillin; AP, antimicrobial peptides; ATP, adenosine triphosphate; AU, adamantyl ureas; AZ, azoles; BCECF, 2',7'-bis-(2-carboxyethyl)-5(6)-carboxyfluorescein; BDQ, bedaquiline; BL, β-lactams; BP, biarylpiperazines; BSP, bisanilinopyrimidines; CFZ, clofazimine; CHL, chloramphenicol; CMB, carboxymycobactins; DAU, daunorubicin; DOX, doxorubicin; EMB, ethambutol; ERY, erythromycin; EtBr, ethidium bromide; FQs, fluoroquinolones; IA, indoleamides; INH, isoniazid; KAN, kanamycin; MB, mycobactins; MFS, major facilitator superfamily; NOR, norfloxacin; NOV, novobiocin; PA, pyrazolones; PD, pyridines; PMF, proton motive force; PR, pyrroles; PUR, puromycin; PY, pyronin Y; RIF, rifampicin; RND, resistance nodulation division; SMR, small multidrug resistance; SPE, spectinomycin; SKI, imidazoline SKI-356313; STR, streptomycin; TET, tetracycline; TPP, tetraphenylphosphonium; VAN, vancomycin. BM212 is a 1,5-diarylpyrrole; SQ109 is an ethylenediamine derivative of ethambutol.
Transporters in bold-type letter corresponded to those evaluated in the present study.

References

1. Aínsa, J.A., Blokpoel, M.C., Otal, I., Young, D.B., De Smet, K.A., and Martín, C. (1998). Molecular cloning and characterization of Tap, a putative multidrug efflux pump present in *Mycobacterium fortuitum* and *Mycobacterium tuberculosis*. J. Bacteriol. 80, 5836-5843.

2. Alland, D., Kramnik, I., Weisbrod, T., Otsubo, L., Cerny, R., Miller, L., et al. (1998). Identification of differentially expressed mRNA in prokaryotic organisms by customized amplification libraries (DECAL): the effect of isoniazid on gene expression in *Mycobacterium tuberculosis*. Proc. Natl. Acad. Sci. U.S.A. 95, 13227-13232.

3. Balganesh, M., Dinesh, N., Sharma, S., Kuruppath, S., Nair, A.V., and Sharma, U. (2012). Efflux pumps of *Mycobacterium tuberculosis* play a significant role in antituberculosis activity of potential drug candidates. Antimicrob. Agents Chemother. 56, 2643-2651. doi: 10.1128/AAC.06003-11

4. Balganesh, M., Kuruppath, S., Marcel, N., Sharma, S., Nair, A., and Sharma, U. (2010). Rv1218c, an ABC transporter of *Mycobacterium tuberculosis* with implications in drug discovery. Antimicrob. Agents Chemother. 54, 5167-5172. doi: 10.1128/AAC.00610-10

5. Banerjee, S.K., Bhatt, K., Rana, S., Misra, P., and Chakraborti, P.K. (1996). Involvement of an efflux system in mediating high level of fluoroquinolone resistance in *Mycobacterium smegmatis*. Biochem. Biophys. Res. Commun. 226, 362-368. doi: 10.1006/bbrc.1996.1362

6. Bianco, M.V., Blanco, F.C., Imperiale, B., Forrellad, M.A., Rocha, R.V., Klepp, L.I., et al. (2011). Role of P27-P55 operon from *Mycobacterium tuberculosis* in the resistance to toxic compounds. BMC Infect. Dis. 11, 195. doi: 10.1186/1471-2334-11-195

7. Braibant, M., and Gilot, P. (2000). The ATP binding cassette (ABC) transport systems of *Mycobacterium tuberculosis*. FEMS Microbiol. Rev. 24, 449-467. doi: 10.1111/j.1574-6976.2000.tb00550.x

8. Braibant, M., Lefevre, P., Dewit, L., Peirs, P., Ooms, J., Hugyen, K., et al. (1996). A *Mycobacterium tuberculosis* gene cluster encoding proteins of a phosphate transporter homologous to the *E. coli* Pst system. Gene. 176, 171-176. doi: 10.1016/0378-1119(96)00242-9

9. Choudhuri, B.S., Bhakta, S., Barik, R., Joyoti, B., Kundu, M., and Chakrabarti, P. (2002). Overexpression and functional characterization of an ABC (ATP-binding cassette) transporter encoded by the genes *drrA* and *drrB* of *Mycobacterium tuberculosis*. Biochem. J. 367, 279-285. doi: 10.1042/bj20020615

10. Colangeli, R., Helb, D., Sridharan, S., Sun, J., Varma-Basil, M., Hazbon, M.H., et al. (2005). The *Mycobacterium tuberculosis iniA* gene is essential for activity of an efflux pump that confers drug tolerance to both isoniazid and ethambutol. Mol. Microbiol. 55, 1829-1840.

11. Da Silva, P., Bigi, F., Santangelo, M., Romano, M., Martín, C., Cataldi, A., et al. (2001). Characterization of P55, a multidrug efflux pump in *Mycobacterium bovis* and *Mycobacterium tuberculosis*. Antimicrob. Agents Chemother. 45, 800-804. doi: 10.1128/AAC.45.3.800-804.2001

12. Danilchanka, O., Mailaender, C., and Niederweis, M. (2008). Identification of a novel multidrug efflux pump of *Mycobacterium tuberculosis*. Antimicrob. Agents Chemother. 52, 2503-2511. doi: 10.1128/AAC.00298-08

13. de Knegt, G.J., Bruning, O., Marian, T., de Jong, M., van Belkum, A., Endtz, H.P., et al. (2013). Rifampicin-induced transcriptome response in rifampicin-resistant *Mycobacterium tuberculosis*. Tuberculosis. 93, 96-101. doi: 10.1016/j.tube.2012.10.013

14. De Rossi, E., Arrigo, P., Bellinzoni, M., Silva, P., Martin, C., Ainsa, J., et al. (2002). The multidrug transporters belonging to major facilitator superfamily in *Mycobacterium tuberculosis*. Mol. Med. 8, 714-724.

15. De Rossi, E., Branzoni, M., Cantoni, R., Milano, A., Riccardi, G., and Ciferri, O. (1998). *mmr*, a *Mycobacterium tuberculosis* gene conferring resistance to small cationic dyes and inhibitors. J. Bacteriol. 180, 6068-6071.

16. Dinesh, N., Sharma, S., and Balganesh, M. (2013). Involvement of efflux pumps in the resistance to peptidoglycan synthesis inhibitors in *Mycobacterium tuberculosis*. Antimicrob. Agents Chemother. 57, 1941-1943. doi: 10.1128/AAC.01957-12

17. Domenech, P., Kobayashi, H., LeVier, K., Walker, G.C., and Barry, C.E. (2009). BacA, an ABC transporter involved in maintenance of chronic murine infections with *Mycobacterium tuberculosis*. J. Bacteriol. 191, 477-485. doi: 10.1128/JB.01132-08

18. Domenech, P., Reed, M., and Barry, C 3rd. (2005). Contribution of the *Mycobacterium tuberculosis* MmpL protein family to virulence and drug resistance. Infect. Immun. 73, 3492-3501. doi: 10.1128/IAI.73.6.3492-3501.2005

19. Doran, J.L., Pang, Y., Mdluli, K.E., Moran, A.J., Victor, T.C., Stokes, R.W., et al. (1997). *Mycobacterium tuberculosis efpA* encodes an efflux protein of the QacA transporter family. Clin. Diagn. Lab. Immunol. 4, 23-32.

20. Gupta, A.K., Katoch, V.M., Chauhan, D.S., Sharma, R., Singh M, Venkatesan, K., et al. (2010). Microarray analysis of efflux pump genes in multidrug-resistant *Mycobacterium tuberculosis* during stress induced by common anti-tuberculous drugs. Microb. Drug Resist. 16, 21-28. doi:10.1089/mdr.2009.0054.

21. Gupta, A.K., Reddy, V.P., Lavania, M., Chauhan, D.S., Venkatesan, K., Sharma, V.D., et al. (2010). *jefA* (Rv2459), a drug efflux gene in *Mycobacterium tuberculosis* confers resistance to isoniazid and ethambutol. Indian J. Med. Res. 132, 176-188.

22. Hao, P., Shi-Liang, Z., Ju, L., Ya-Xin, D., Biao, H., Xu, W., et al. (2011). The role of ABC efflux pump, Rv1456c-Rv1457c-Rv1458c, from *Mycobacterium tuberculosis* clinical isolates in China. Folia Microbiol. 56, 549-553. doi: 10.1007/s12223-011-0080-7

23. Harris, K.K., Fay, A., Yan, G.H., Kunwar, P., Socci, N.D., Pottabathini, N., et al. (2014). Novel imidazoline antimicrobial scaffold that inhibits DNA replication with activity against mycobacteria and drug resistant Gram-positive cocci. ACS Chem. Biol. 9, 2572-2583. doi: 10.1021/cb500573z

24. Hartkoorn, R., Uplekar, S., and Cole, S. (2014). Cross-resistance between clofazimine and bedaquiline through upregulation of MmpL5 in *Mycobacterium tuberculosis*. Antimicrob. Agents Chemother. 58, 2979-2981. doi: 10.1128/AAC.00037-14

25. Jiang, X., Zhang, W., Zhang, Y., Gao, F., Lu, C., Zhang, X., et al. (2008). Assessment of efflux pump gene expression in a clinical isolate *Mycobacterium tuberculosis* by real-time reverse transcription PCR. Microb. Drug Resist. 14, 7-11. doi:10.1089/mdr.2008.0772.

26. La Rosa, V., Poce, G., Canseco, J.O., Buroni, S., Pasca, M.R., Biava, M., et al. (2012). MmpL3 is the cellular target of the antitubercular pyrrole derivative BM212. Antimicrob. Agents Chemother. 56, 324-331. doi: 10.1128/AAC.05270-11

27. Li, G., Zhang, J., Guo, Q., Jiang, Y., Wei, J., Zhao, L.L., et al. (2015). Efflux pump gene expression in multidrug-resistant *Mycobacterium tuberculosis* clinical isolates. PloS One. 10, e0119013. doi: 10.1371/journal.pone.0119013

28. Li, W., Upadhyay, A., Fontes, F.L., North, E.J., Wang, Y., Crans, D.C., et al. (2014). Novel insights into the mechanism of inhibition of MmpL3, a target of multiple pharmacophores in *Mycobacterium tuberculosis*. Antimicrob. Agents Chemother. 58, 6413-6423. doi: 10.1128/AAC.03229-14

29. Lu, J., Liu, M., Wang, Y., Pang, Y., and Zhao, Z. (2014). Mechanisms of fluoroquinolone monoresistance in *Mycobacterium tuberculosis*. FEMS Microbiol. Lett. 353, 40-48. doi: 10.1111/1574-6968.12401

30. Machado, D., Couto, I., Perdigão, J., Rodrigues, L., Baptista, P., Portugal, I., et al. (2012). Contribution of efflux to the emergence of isoniazid and multidrug resistance in *Mycobacterium tuberculosis*. PLoS One. 7, e34538. doi: 10.1371/journal.pone.0034538

31. Milano, A., Pasca, M., Provvedi, R., Lucarelli, A., Manina, G., Ribeiro, A., et al. (2009). Azole resistance in *Mycobacterium tuberculosis* is mediated by the MmpS5–MmpL5 efflux system. Tuberculosis. 89, 84-90. doi: 10.1016/j.tube.2008.08.003.

32. Pang. Y., Lu, J., Wang, Y., Song, Y., Wang, S., and Zhao, Y. (2013). Study of the rifampin monoresistance mechanism in *Mycobacterium tuberculosis*. Antimicrob. Agents Chemother. 5, 893-900. doi: 10.1128/AAC.01024-12

33. Pasca, M., Guglierame, P., Arcesi, F., Bellinzoni, M., De Rossi, E., and Riccardi, G. (2004). Rv2686c-Rv2687c-Rv2688c, an ABC fluoroquinolone efflux pump in *Mycobacterium tuberculosis*. Antimicrob. Agents Chemother. 48, 3175-3178. doi: 10.1128/AAC.48.8.3175-3178.2004

34. Pasca, M., Guglierame, P., De Rossi, E., Zara, F., and Riccardi, G. (2005). *mmpL7* gene of *Mycobacterium tuberculosis* is responsible for isoniazid efflux in *Mycobacterium smegmatis*. Antimicrob. Agents Chemother. 49, 4775-4777. doi: 10.1128/AAC.49.11.4775-4777.2005

35. Ramón-García, S., Martín, C., Thompson C., and Aínsa J. (2009). Role of the *Mycobacterium tuberculosis* P55 efflux pump in intrinsic drug resistance, oxidative stress responses, and growth. Antimicrob. Agents Chemother. 53, 3675-3682. doi: 10.1128/AAC.00550-09

36. Ramón-García, S., Martín, C., De Rossi, E., and Aínsa, J. (2007). Contribution of the Rv2333c efflux pump (the Stp protein) from *Mycobacterium tuberculosis* to intrinsic antibiotic resistance in *Mycobacterium bovis* BCG. J. Antimicrob. Chemother. 59, 544-547. doi: 10.1093/jac/dkl510

37. Ramón-García, S., Mick, V., Dainese, E., Martín, C., Thompson, C., De Rossi, E., et al. (2012). Functional and genetic characterization of the Tap efflux pump in *Mycobacterium bovis* BCG. Antimicrob. Agents Chemother. 56, 2074-2083.

38. Rodrigues, L., Villellas, C., Bailo, R., Viveiros, M., and Aínsa, J. (2013). Role of the Mmr efflux pump in drug resistance in *Mycobacterium tuberculosis*. Antimicrob. Agents Chemother. 57, 751-757. doi: 10.1128/AAC.01482-12

39. Sarin, J., Aggarwal, S., Chaba, R., Varshney, G.C., and Chakraborti, P.K. (2001). β-subunit of phosphate-specific transporter from *Mycobacterium tuberculosis* is a thermostable ATPase. J. Biol. Chem. 276, 44590-44597. doi: 10.1074/jbc.M105401200

40. Siddiqi, N., Das, R., Pathak, N., Banerjee, S., Ahmed, N., Katoch, V., et al. 2004. *Mycobacterium tuberculosis* isolate with a distinct genomic identity overexpresses a tap-like efflux pump. Infection. 32, 109-111. doi: 10.1007/s15010-004-3097-x

41. Spivey, V.L., Whalan, R.H., Hirst, E.M., Smerdon, S.J., and Buxton, R.S. (2013). An attenuated mutant of the Rv1747 ATP-binding cassette transporter of *Mycobacterium tuberculosis* and a mutant of its cognate kinase, PknF, show increased expression of the efflux pump-related *iniBAC* operon. FEMS Microbiol Lett. 347, 107-115. doi: 10.1111/1574-6968.12230

42. Tahlan, K., Wilson, R., Kastrinsky, D.B., Arora, K., Nair, V., Fischer, E., et al. (2012). SQ109 targets MmpL3, a membrane transporter of trehalose monomycolate involved in mycolic acid donation to the cell wall core of *Mycobacterium tuberculosis*. Antimicrob. Agents Chemother. 56, 1797-1809. doi: 10.1128/AAC.05270-11

43. Wang, K., Pei, H., Huang, B., Zhu, X., Zhang, J., Zhou, B., et al. (2013). The expression of ABC efflux pump, Rv1217c–Rv1218c, and its association with multidrug resistance of *Mycobacterium tuberculosis* in China. Curr. Microbiol. 66, 222-226. doi: 10.1007/s00284-012-0215-3

44. Wells, R.M., Jones, C.M., Xi, Z., Speer, A., Danilchanka, O., Doornbos, K.S., et al. (2013). Discovery of a siderophore export system essential for virulence of *Mycobacterium tuberculosis*. PLoS Pathog. 9, e1003120. doi: 10.1371/journal.ppat.1003120
