# Supplementary material for: Interplay between Mutations and Efflux in Drug Resistant Clinical Isolates of Mycobacterium tuberculosis
Source: Front Microbiol. 2017 Apr 27;8:711. doi: 10.3389/fmicb.2017.00711 (PMC5406451; doi:10.3389/fmicb.2017.00711)
Supplement: Supplementary file 2 [file Table2.PDF]

# *Supplementary Material*

# Interplay between mutations and efflux in drug resistant *Mycobacterium tuberculosis* clinical isolates

**Diana Machado[1§], Tatiane Coelho[2,3§], João Perdigão[4], Catarina Pereira[4], Isabel Couto[1], Isabel Portugal[4], Raquel Maschmann[2,5], Daniela Ramos[3], Andrea von Groll[3], Maria Lúcia Rossetti[5,6], Pedro A. Silva[2,3]† and Miguel Viveiros[1]†***

*** Correspondence:** Miguel Viveiros: mviveiros@ihmt.unl.pt

## 1 Supplementary Table

**Supplementary Table 2. Enhancement of the inhibitory activity of antibiotics by the efflux inhibitors towards the *M. tuberculosis* strain MtbPT11 (XDR) determined by quantitative susceptibility testing.**

| Combination | qDST | TTD (days; hours) | ΔTTD (hours) | Potentiation of EIs on ATB activity (%) |
|---|---|---|---|---|
| ***MtbPT11 – XDR*** | | | | |
| Non-exposed | - | 5;3 | - | - |
| VP 128 µg/ml | R | 5;09 | - | - |
| TZ 7.5 µg/ml | R | 4;1 | - | - |
| CPZ 15 µg/ml | R | 6;07 | - | - |
| INH 1 µg/ml | R | 6;6 | - | - |
| INH 1 µg/ml + VP | **S** | **9;11** | **77** | **38.03** |
| INH 1 µg/ml + TZ | R | 7;05 | 18 | 6.82 |
| INH 1 µg/ml + CPZ | **S** | **10;17** | **107** | **54.09** |
| INH 0.1 µg/ml | R | 3;12 | - | - |
| INH 0.1 µg/ml + VP | R | **5;1** | **37** | **63;46** |
| INH 0.1 µg/ml + TZ | R | 4;12 | 24 | 32;05 |
| INH 0.1 µg/ml + CPZ | R | **5;05** | **36** | **61;86** |
| RIF 20 µg/ml | R | 4;06 | | |
| RIF 20 µg/ml + VP | **S** | **20;04** | **386** | **393.60** |
| RIF 20 µg/ml + TZ | R | **6;12** | **60** | **50.74** |
| RIF 20 µg/ml + CPZ | **S** | **12;09** | **195** | **197.78** |
| RIF 4 µg/ml | R | 3;12 | - | - |
| RIF 4 µg/ml + VP | R | **7;01** | **79** | **121.14** |
| RIF 4 µg/ml + TZ | R | **50;8** | **31** | **60.25** |
| RIF 4 µg/ml + CPZ | R | **6;16** | **71** | **94.32** |
| RIF 1 µg/ml | R | 3;1 | - | - |
| RIF 1 µg/ml + VP | R | **6;1** | **72** | **96.77** |
| RIF 1 µg/ml + TZ | R | **5;06** | **48** | **63.23** |
| RIF 1 µg/ml + CPZ | R | **6;07** | **72** | **95.81** |
| AMK 20 µg/ml | R | 3;18 | - | - |
| AMK 20 µg/ml + VP | R | **5;05** | **45** | **61.95** |
| AMK 20 µg/ml + TZ | R | **5;02** | **30** | **57.86** |
| AMK 20 µg/ml + CPZ | R | **5;13** | **43** | **61.32** |
| AMK 4 µg/ml | R | 4;01 | - | - |

| | | | | |
|---|---|---|---|---|
| AMK 4 μg/ml + VP | R | **6;1** | **48** | **52.12** |
| AMK 4 μg/ml + TZ | R | 4;19 | 19 | 4.49 |
| AMK 4 μg/ml + CPZ | R | 5;22 | 46 | 30.17 |
| AMK 1 μg/ml | R | 3;06 | - | - |
| AMK 1 μg/ml + VP | R | **6;02** | **72** | **96.73** |
| AMK 1 μg/ml + TZ | R | 4;19 | 43 | 36.93 |
| AMK 1 μg/ml + CPZ | R | **6;02** | **72** | **96.73** |
| CAP 25 μg/ml | R | **6;12** | - | - |
| CAP 25 μg/ml + VP | **S** | **61;16** | **1324** | **899.35** |
| CAP 25 μg/ml + TZ | **S** | **43;1** | **1040** | **604.25** |
| CAP 25 μg/ml + CPZ | S | **63;1** | **1405** | **931.05** |
| CAP 5 μg/ml | R | **4;06** | - | - |
| CAP 5 μg/ml + VP | **S** | **13;19** | **331** | **224.88** |
| CAP 5 μg/ml + TZ | R | **10;16** | **160** | **150.25** |
| CAP 5 μg/ml + CPZ | **S** | **41;23** | **1007** | **915.52** |
| CAP 2.5 μg/ml | R | **3;23** | - | - |
| CAP 2.5 μg/ml + VP | R | **6;2** | **51** | **91.95** |
| CAP 2.5 μg/ml + TZ | R | **5;13** | **38** | **58.82** |
| CAP 2.5 μg/ml + CPZ | R | **6;19** | **68** | **91.64** |
| OFX 2 μg/ml | R | 3;15 | - | - |
| OFX 2 μg/ml + VP | R | **6;22** | **79** | **97.46** |
| OFX 2 μg/ml + TZ | R | **5;02** | **57** | **59.37** |
| OFX 2 μg/ml + CPZ | R | **6;16** | **73** | **95.56** |
| OFX 1 μg/ml | R | 3;08 | - | - |
| OFX 1 μg/ml + VP | R | **6;01** | **72** | **95.13** |
| OFX 1 μg/ml + TZ | R | 4;23 | 47 | 37.34 |
| OFX 1 μg/ml + CPZ | R | **5;19** | **67** | **68.51** |

ATB, antibiotic; AMK, amikacin; CAP, capreomycin; CPZ, chlorpromazine; EI, efflux inhibitor; INH, isoniazid; OFX, ofloxacin; qDST, quantitative drug susceptibility testing; R, resistant; RIF, rifampicin; S, susceptible; TZ, thioridazine; VP, verapamil; XDR, extensively drug resistant.

TTD, time to detection; ΔTTD, variation of time to detection of growth between the tube containing the antibiotic plus EI and the tube with only the antibiotic.

Values in bold corresponded to enhancement of antibiotic activity equal or above 50%.
