# Supplementary material for: Interplay between Mutations and Efflux in Drug Resistant Clinical Isolates of Mycobacterium tuberculosis
Source: Front Microbiol. 2017 Apr 27;8:711. doi: 10.3389/fmicb.2017.00711 (PMC5406451; doi:10.3389/fmicb.2017.00711)
Supplement: Supplementary file 3 [file Table3.PDF]

Supplementary Material

Interplay between mutations and efflux in drug resistant *Mycobacterium tuberculosis* clinical isolates

Diana Machado[1§], Tatiane Coelho[2,3§], João Perdigão[4], Catarina Pereira[4], Isabel Couto[1], Isabel Portugal[4], Raquel Maschmann[2,5], Daniela Ramos[3], Andrea von Groll[3], Maria Lúcia Rossetti[5,6], Pedro A. Silva[2,3]† and Miguel Viveiros[1]†*

*** Correspondence:** Miguel Viveiros: mviveiros@ihmt.unl.pt

1 Supplementary Table

Supplementary Table 3. Enhancement of the inhibitory activity of antibiotics by the efflux inhibitors verapamil, thioridazine and chlorpromazine towards the *M. tuberculosis* strains MtbPT3 (INH^R) and MtbPT5 (RIF^R) determined by quantitative susceptibility testing.

Combination	qDST	TTD (days; hours)	ΔTTD (hours)	Potentiation of EIs on ATB activity (%)
MtbPT3 - INH^R				
Non-exposed	-	3;10	-	-
VP 128 µg/ml	R	3;2	-	-
TZ 7.5 µg/ml	R	3;14	-	-
CPZ 15 µg/ml	R	5;04	-	-
INH 3 µg/ml	R	4;18	-	-
INH 3 µg/ml + VP	R	**6;23**	**53**	**50.04**
INH 3 µg/ml + TZ	**S**	**10;04**	**126**	**140.19**
INH 3 µg/ml + CPZ	**S**	**13;04**	**200**	**215.79**
INH 1 µg/ml	R	3;07	-	-
INH 1 µg/ml + VP	R	4;08	25	32.90
INH 1 µg/ml + TZ	R	3;02	-24	-1.63
INH 1 µg/ml + CPZ	R	**5;02**	**48**	**63.52**
INH 0.1 µg/ml	R	3;07	-	-
INH 0.1 µg/ml + VP	R	4;08	25	32.90
INH 0.1 µg/ml + TZ	R	3;1	10	0.98
INH 0.1 µg/ml + CPZ	R	4;19	43	36.48
MtbPT5 - RIF^R				
Non-exposed		3;16	-	-
VP 128 µg/ml	R	4;20	-	-
TZ 7.5 µg/ml	R	5:1	-	-
CPZ 15 µg/ml	R	4;11	-	-
RIF 20 µg/ml	R	4;12	-	-
RIF 20 µg/ml + VP	R	**7;12**	**62**	**74.76**
RIF 20 µg/ml + TZ	R	6;1	37	48.06
RIF 20 µg/ml + CPZ	R	6;15	51	49.27
RIF 4 µg/ml	R	3;14	-	**-**

RIF 4 µg/ml + VP	R	4;21	31	34.08
RIF 4 µg/ml + TZ	R	**5;11**	**45**	**62.74**
RIF 4 µg/ml + CPZ	R	**6;19**	**77**	**97.13**
RIF 1 µg/ml	R	3;14	-	-
RIF 1 µg/ml + VP	R	4;22	32	34.39
RIF 1 µg/ml + TZ	R	4;15	25	32.17
RIF 1 µg/ml + CPZ	R	**6;8**	**78**	**116.56**

ATB, antibiotic; CPZ, chlorpromazine; EI, efflux inhibitor; INH, isoniazid; qDST, quantitative drug susceptibility testing; R, resistant; RIF, rifampicin; S, susceptible; TZ, thioridazine; VP, verapamil.
TTD, time to detection; ΔTTD, variation of time to detection of growth between the tube containing the antibiotic plus EI and the tube with only the antibiotic.
Values in bold corresponded to enhancement of antibiotic activity equal or above 50%.
