# Supplementary material for: Interplay between Mutations and Efflux in Drug Resistant Clinical Isolates of Mycobacterium tuberculosis
Source: Front Microbiol. 2017 Apr 27;8:711. doi: 10.3389/fmicb.2017.00711 (PMC5406451; doi:10.3389/fmicb.2017.00711)
Supplement: Supplementary file 4 [file Table4.PDF]

Supplementary Material

Interplay between mutations and efflux in drug resistant *Mycobacterium tuberculosis* clinical isolates

Diana Machado[1§], Tatiane Coelho[2,3§], João Perdigão[4], Catarina Pereira[4], Isabel Couto[1], Isabel Portugal[4], Raquel Maschmann[2,5], Daniela Ramos[3], Andrea von Groll[3], Maria Lúcia Rossetti[5,6], Pedro A. Silva[2,3]† and Miguel Viveiros[1]†*

*** Correspondence:** Miguel Viveiros: mviveiros@ihmt.unl.pt

1 Supplementary Table

Supplementary Table 4. Enhancement of the inhibitory activity of antibiotics by the efflux inhibitors verapamil, thioridazine and chlorpromazine towards the *M. tuberculosis* strains MtbBZ1 (OFX^R) and MtbPT7 (MDR) determined by quantitative susceptibility testing.

Combination	qDST	TTD (days; hours)	ΔTTD (hours)	Potentiation of EIs on ATB activity (%)
MtbBZ1 - OFX^R				
Non-exposed	-	3;17	-	-
VP 128 µg/ml	R	5;5	-	-
TZ 7.5 µg/ml	R	5;1	-	-
CPZ 15 µg/ml	R	5;25	-	-
OFX 10 µg/ml	R	6;5	-	-
OFX 10 µg/ml + VP	R	6;5	0	0
OFX 10 µg/ml + TZ	R	7;7	26	18.16
OFX 10 µg/ml + CPZ	R	8;8	51	35
OFX 2 µg/ml	R	3;17	-	-
OFX 2 µg/ml + VP	R	**7;17**	**96**	**126.18**
OFX 2 µg/ml + TZ	R	4;2	9	32.49
OFX 2 µg/ml + CPZ	R	**6;15**	**70**	**94.01**
OFX 1 µg/ml	R	3;15	-	-
OFX 1 µg/ml + VP	R	**7;5**	**96**	**126.98**
OFX 1 µg/ml + TZ	R	4;21	30	33.65
OFX 1 µg/ml + CPZ	R	**6;14**	**71**	**94.92**
MtbPT7 - MDR				
Non-exposed	-	3;19	-	-
VP 128 µg/ml	R	4;07	-	-
TZ 7.5 µg/ml	R	4;07	-	-
CPZ 15 µg/ml	R	4;16	-	-
INH 1 µg/ml	R	9;07	-	-
INH 1 µg/ml + VP	**S**	10;02	25	10.47
INH 1 µg/ml + TZ	**S**	11;18	71	23.26
INH 1 µg/ml + CPZ	**S**	**18;08**	**216**	**99.34**
INH 0.1 µg/ml	R	3;12	-	-

INH 0.1 µg/ml + VP	R	4;12	24	32.05
INH 0.1 µg/ml + TZ	R	4;07	12	30.45
INH 0.1 µg/ml + CPZ	R	**5;08**	**36**	**62.82**
RIF 20 µg/ml	R	4;22	-	-
RIF 20 µg/ml + VP	**S**	**33**	**674**	**681.99**
RIF 20 µg/ml + TZ	R	4;16	-6	-1.42
RIF 20 µg/ml + CPZ	R	5;22	24	23.70
RIF 4 µg/ml	R	4;04	-	-
RIF 4 µg/ml + VP	R	5;06	26	25.25
RIF 4 µg/ml + TZ	R	3;23	-26	-20.05
RIF 4 µg/ml + CPZ	R	4;14	14	2.48
RIF 1 µg/ml	R	3;13	-	-
RIF 1 µg/ml + VP	R	4;14	25	32.27
RIF 1 µg/ml + TZ	R	3;18	5	1.60
RIF 1 µg/ml + CPZ	R	4;1	12	30.99

ATB, antibiotic; CPZ, chlorpromazine; EI, efflux inhibitor; INH, isoniazid; OFX, ofloxacin; qDST, quantitative drug susceptibility testing; R, resistant; RIF, rifampicin; S, susceptible; TZ, thioridazine; VP, verapamil.

TTD, time to detection; ΔTTD, variation of time to detection of growth between the tube containing the antibiotic plus EI and the tube with only the antibiotic.

Values in bold-type letter corresponded to enhancement of antibiotic activity equal or above 50%.
